# Increased Histone H3 Phosphorylation in Neurons in Specific Brain Structures after Induction of Status Epilepticus in Mice

**DOI:** 10.1371/journal.pone.0077710

**Published:** 2013-10-16

**Authors:** Tetsuji Mori, Taketoshi Wakabayashi, Haruyuki Ogawa, Yukie Hirahara, Taro Koike, Hisao Yamada

**Affiliations:** Department of Anatomy and Cell Science, Kansai Medical University, Hirakata, Osaka, Japan; Osaka University Graduate School of Medicine, Japan

## Abstract

Status epilepticus (SE) induces pathological and morphological changes in the brain. Recently, it has become clear that excessive neuronal excitation, stress and drug abuse induce chromatin remodeling in neurons, thereby altering gene expression. Chromatin remodeling is a key mechanism of epigenetic gene regulation. Histone H3 phosphorylation is frequently used as a marker of chromatin remodeling and is closely related to the upregulation of mRNA transcription. In the present study, we analyzed H3 phosphorylation levels *in vivo* using immunohistochemistry in the brains of mice with pilocarpine-induced SE. A substantial increase in H3 phosphorylation was detected in neurons in specific brain structures. Increased H3 phosphorylation was dependent on neuronal excitation. In particular, a robust upregulation of H3 phosphorylation was detected in the caudate putamen, and there was a gradient of phosphorylated H3^+^ (PH3^+^) neurons along the medio-lateral axis. After unilateral ablation of dopaminergic neurons in the substantia nigra by injection of 6-hydroxydopamine, the distribution of PH3^+^ neurons changed in the caudate putamen. Moreover, our histological analysis suggested that, in addition to the well-known MSK1 (mitogen and stress-activated kinase)/H3 phosphorylation/c-fos pathway, other signaling pathways were also activated. Together, our findings suggest that a number of genes involved in the pathology of epileptogenesis are upregulated in PH3^+^ brain regions, and that H3 phosphorylation is a suitable indicator of strong neuronal excitation.

## Introduction

Temporal lobe epilepsy (TLE) is the most common type of epilepsy. The animal model of TLE can be created by administration of pilocarpine, a muscarinic acetylcholine receptor agonist. Administration of pilocarpine in experimental animals induces status epilepticus (SE), followed by a seizure-free latent phase lasting for several weeks. In general, diazepam is administrated to reduce mortality several hours after pilocarpine. Those animals subsequently develop spontaneous recurrent seizure without remission. Accordingly, pathological changes in the brain after SE are critical for understanding the process of epileptogenesis [1]. SE induces morphological and pathological changes in the brain, such as mossy fiber sprouting in the hippocampus, inducing proliferation of neural precursors in the dentate gyrus of the hippocampus (DG) and the subventricular zone (SVZ), and neuronal cell death in discrete regions [2-4]. These morphological and pathological changes are associated with altered gene expression. Recently, the epigenetic control of gene expression has received increasing attention. Chromatin remodeling is an epigenetic mechanism regulating gene expression.

Chromatin is composed of DNA and hisotones. Histones include H2A, H2B, H3 and H4. The N-terminals of the various histones are highly conserved from yeast to mammals, and are modified by phosphorylation, acetylation and methylation [5]. These modifications are a critical step in chromatin remodeling, resulting in the regulation of gene expression. In general, histone phosphorylation and acetylation are associated with transcriptional activation, while methylation is associated with transcriptional repression [5,6]. The hippocampus is a brain region characterized by extensive neuroplasticity. Here, dynamic processes associated with learning and memory formation are active, including synaptogenesis, long-term potentiation, dendritic remodeling and neurogenesis. Recently, it has been suggested that chromatin remodeling in the hippocampal neurons is responsible for learning and memory formation [5].

It is well known that seizures upregulate the expression of various immediate early genes, especially c-fos, which has been studied in detail [7-9]. c-fos expression is regulated by many mechanisms, and accumulating evidence suggests that histone modification is a key mechanism controlling c-fos mRNA expression [5,10,11]. H3 phosphorylation at Ser10 and acetylation at Lys14 are frequently used markers for detecting histone modification [6,12-14]. After seizures, H3 phosphorylation in hippocampal neurons increases, and is followed by an elevation in c-fos expression [12,13]. H3 phosphorylation occurs in the c-fos promoter region in the rat hippocampus after seizures [11].

H3 phosphorylation in neurons in the central nervous system is induced by activation of NMDA receptors through light and stress [14-16]. H3 phosphorylation in neurons after induction of seizures has been well characterized in the hippocampus *in vivo* [12-14,17]. However, information on H3 phosphorylation in other brain regions is lacking. An *in vitro* study revealed that activation of NMDA receptors induces H3 phosphorylation in cultured striatal neurons [16]. Dopaminergic terminals (of neurons in the substantia nigra) are densely present in the caudate putamen (CPu) and the nucleus accumbens (Acb). Activation of dopamine D1 receptor induces H3 phosphorylation in neurons in the CPu [18-21]. Blocking dopamine D2 and related receptors with haloperidol (an anti-psychotic drug) also induces H3 phosphorylation, through both the c-AMP/PKA and NMDA receptor pathways [17]. From these literatures, one can infer that H3 phosphorylation also occurs in neurons outside of the hippocampus *in vivo* after SE, but there has been no detailed analysis. Clarifying distribution of phosphorylated H3 immunopositive (PH3^+^) neurons in the SE brain should be useful for understanding of pathology of epileptogenesis. 

In the present study, we analyzed the distribution of PH3^+^ cells in a mouse model of pilocarpine-induced SE. Our findings suggest that H3 phosphorylation is restricted to selective brain structures after SE, and that dopaminergic tone, from the midbrain to the forebrain, has a crucial role in the process.

## Materials and Methods

### Animals

Six- or seven-week-old male ICR mice were used in all experiments. Mice were supplied by Japan SLC. Inc. (Hamamatsu, Japan). The experimental protocols were approved by the animal ethics committee at Kansai Medical University. Stereotaxis coordinates of the brain are based on those of Paxinos and Franklin (2001).

### Drug treatment of mice

Pilocarpine-induced SE model mice were created as follows. Scopolamine methyl bromide (scopolamine; SIGMA, St. Louis, MO, USA) and pilocarpine hydrochloride (pilocarpine; Tokyo Kasei, Tokyo, Japan) were dissolved in phosphate-buffered saline (PBS) at 0.2 mg/ml and 50 mg/ml, respectively. Diazepam (Wako, Tokyo, Japan) was dissolved in 40% propylene glycol/10% ethanol at 1 mg/ml. MK-801 (Wako), an antagonist of NMDA receptors, was dissolved in PBS at 0.5 mg/ml. All drugs were administered intraperitoneally. Scopolamine was administered at a dose of 1 mg/kg to prevent peripheral responses to pilocarpine. Pilocarpine was administered at a dose of 300 mg/kg 30 min after scopolamine. An identical volume of scopolamine and PBS was administered to control mice. Behavior of mice was observed for 30 min after pilocarpine administration. Most mice started to exhibit myoclonus within 10 min. Seizures developed to continuous tremor and finally to generalized clonic seizures with falling that lasted for several seconds. Subsequently, mice exhibited continuous wet-dog shakes and periodical generalized clonic seizures. Mice that exhibited milder behavioral change (no response, sniffing, head-nodding, myoclonus or a single attack of generalized clonic seizures without continuous wet-dog shakes) were not used for analysis. To stop seizures, diazepam was administered at a dose of 10 mg/kg 1 h after pilocarpine administration. To induce subconvulsive seizure, pilocarpine was administered at a dose of 200 mg/kg. These mice exhibited head-nodding behavior. Mice were fixed at 1, 2, 4 or 8 h after pilocarpine administration. For the 1-h mouse group, diazepam was not administered. Some mice were not administered diazepam to examine the effect of diazepam. In some experiments, MK-801 was administered at a dose of 4 mg/kg. Mice received MK-801 20 min prior to pilocarpine and were fixed 1 h after pilocarpine. Or mice received MK-801 immediately after the first attack of generalized clonic seizure and were fixed 1h after MK-801.

Kainic acid (KA)-induced SE model mice were created as follows. KA (Wako) was dissolved at a concentration of 2 mg/ml in sterilized PBS. Each mouse received unilateral injection of 0.5 μl of KA into the right basolateral amygdala (coordinates: -1.0 mm caudal, 2.8 mm lateral to the bregma, 3.8 mm below the surface of the brain) with a glass capillary over a 2-min period, with a 5-min waiting period before withdrawal, under pentobarbital anesthesia (50 mg/kg). About 1 h after injection of pentobarbital, mice recovered from anesthesia. Mice exhibited SE with repeated generalized clonic seizure and with much more severe epileptic behavior than mice with pilocarpine-induced SE. Mice were fixed 3 h after KA injection.

Ablation of dopaminergic neurons in the substantia nigra was performed as follows. 6-hydroxydopamine (6-OHDA; Wako) was dissolved at a concentration of 3.6 μg/μl in 0.9% NaCl containing 2 mg/ml L-ascorbic acid. Mice were anesthetized with pentobarbital (50 mg/kg), and 6-OHDA was injected unilaterally into the right medial forebrain bundle (coordinates: -1.1 mm anterior, 1.3 mm lateral to the bregma, -4.5 mm below the surface of the brain). A 0.5 μl volume of 6-OHDA solution was injected with a glass capillary over a 2-min period, with a 5-min waiting period before withdrawal. Three weeks after the operation, mice were used for the pilocarpine-induced SE experiments.

### Histological procedures

For histological analysis, mice were deeply anesthetized with pentobarbital (100 mg/kg) and perfused transcardially with PBS, followed by 3% formaldehyde in PBS. The brains were removed, post-fixed with the same fixative overnight, and cryoprotected with 20% sucrose in PBS. Brains were embedded in O.C.T. compound (Sakura Finetek, Tokyo, Japan), snap frozen on dry ice, and cut transversely using a cryostat. Coronal floating sections were made at a thickness of 30 μm. The sections were then processed for immunohistochemistry. To detect PH3, sections were heated at 60 °C in L.A.B. solution (Polysciences, Warrington, PA, USA) for 10 min. To detect Ki67, sections were heated at 99 °C in 0.01 M citrate buffer (pH 6.0) for 10 min. For double-immunostaining with PH3 and another primary antibody, sections were incubated in a primary antibody cocktail. The following primary antibodies were used: rabbit anti-phosphohistone H3 (PH3) (1:600; Upstate, Temecula, CA, USA), rabbit anti-Ki67 (1:1 000; Novocastra, Newcastle, UK), rabbit anti-c-fos (Ab-5; 1:2 000; Millipore, Billerica, MA, USA), rabbit anti-pMSK1 (1:50; Cell Signaling Technology, Danvers, MA, USA), mouse anti-NeuN (1:200; Millipore), goat anti-Iba1 (1:400; Abcam, Cambridge, UK), mouse anti-S100β (1:1 000; SIGMA), goat anti-Olig2 (1:400; Santa Cruz Biotechnology, Santa Cruz, CA, USA), mouse anti-tyrosine hydroxylase (TH) (1:8 000; Invitrogen, Carlsbad, CA, USA). Primary antibodies were detected using species-specific donkey secondary antibodies conjugated to Cy2 or Cy3 (1:200; Jackson ImmunoResearch, West Grove, PA, USA). To visualize nuclei, stained sections were mounted onto glass slides using a medium containing 100 mM DTT, 50% glycerol and 5 μg/ml Hoechst 33258. To detect damaged neurons, Fluoro-Jade C (FJC, Millipore) staining was performed according to the protocol described by the provider. Briefly, coronal floating sections were mounted on the glass slides and were sequentially immersed in the following solutions: 1% NaOH in 80% ethanol for 5 min, 70% ethanol for 2 min, distilled water for 2 min, 0.06% potassium permanganate for 10 min, distilled water for 2 min, 0.0001% of FJC in 0.1% acetic acid for 10 min, and three changes of distilled water for 1 min per changes. The sections were dried and were cleared in xylene and then coverslipped with DPX (SIGMA). Epifluorescence images were acquired with a BZ-9000 microscope (Keyence, Osaka, Japan), and stacked epifluorescence images (three layers with 5-μm intervals) were made. Single optical confocal images were acquired with a LSM510-Meta microscope (Carl Zeiss, Oberkochen, Germany).

### Image analysis

Every tenth coronal section was collected from 1.70 mm to -1.06 mm anteroposterior to the bregma, and in total, nine sections were analyzed from each mouse. Sets of three sections were grouped as (i) anterior, (ii) middle or (iii) posterior, with each set containing (i) the Acb and the anterior CPu, (ii) the middle CPu, the anterior bed nucleus of the stria terminalis (BNST) and the anterior interstitial nucleus of the posterior limb of the anterior commissure (IPAC), (iii) the posterior CPu, the posterior BNST and the posterior IPAC. To compare the density of PH3^+^ cells within the CPu, the CPu was divided into three regions (using two vertical equidistant lines) that were defined as medial, middle and lateral CPu. For analysis of the cortex, every tenth coronal section was collected from 2.00 mm to -1.06 mm anteroposterior to the bregma, and in total, ten sections were analyzed from each mouse. The cortex was divided into three regions: the prelimbic cortex (PL), the cingulate cortex (Cg) and the sensorimotor cortex (SM).

Sections stained with anti-PH3 antibody were scanned at a single layer using a Nanozoomer (Hamamatsu Photonics, Shizuoka, Japan) with a 20× objective lens and automatic focusing function. Scanned images were analyzed to quantify PH3^+^ nuclei with Metamorph (Molecular Devices, Sunnyvale, CA, USA) using the Multiwavelength Cell-Scoring function. All of the microscopy and analysis software settings were unchanged throughout the quantification procedure.

Densities of PH3^+^ and c-fos^+^ cells are presented as the average ± standard error of the mean. Level of significance was determined using the two-tailed unpaired Welch’s *t*-test to compare two groups and the one-way ANOVA followed by Tukey’s post hoc test to compare three groups. At least three mice were analyzed in each group. Number of animals used in each experiment is indicated in each figure legend. Statistical significance was set at *p* < 0.05. Statistical analysis was performed using R, a statistical software package (http://www.R-project.org).

## Results

### SE induces phosphorylation of histone H3

**Figure 1:**
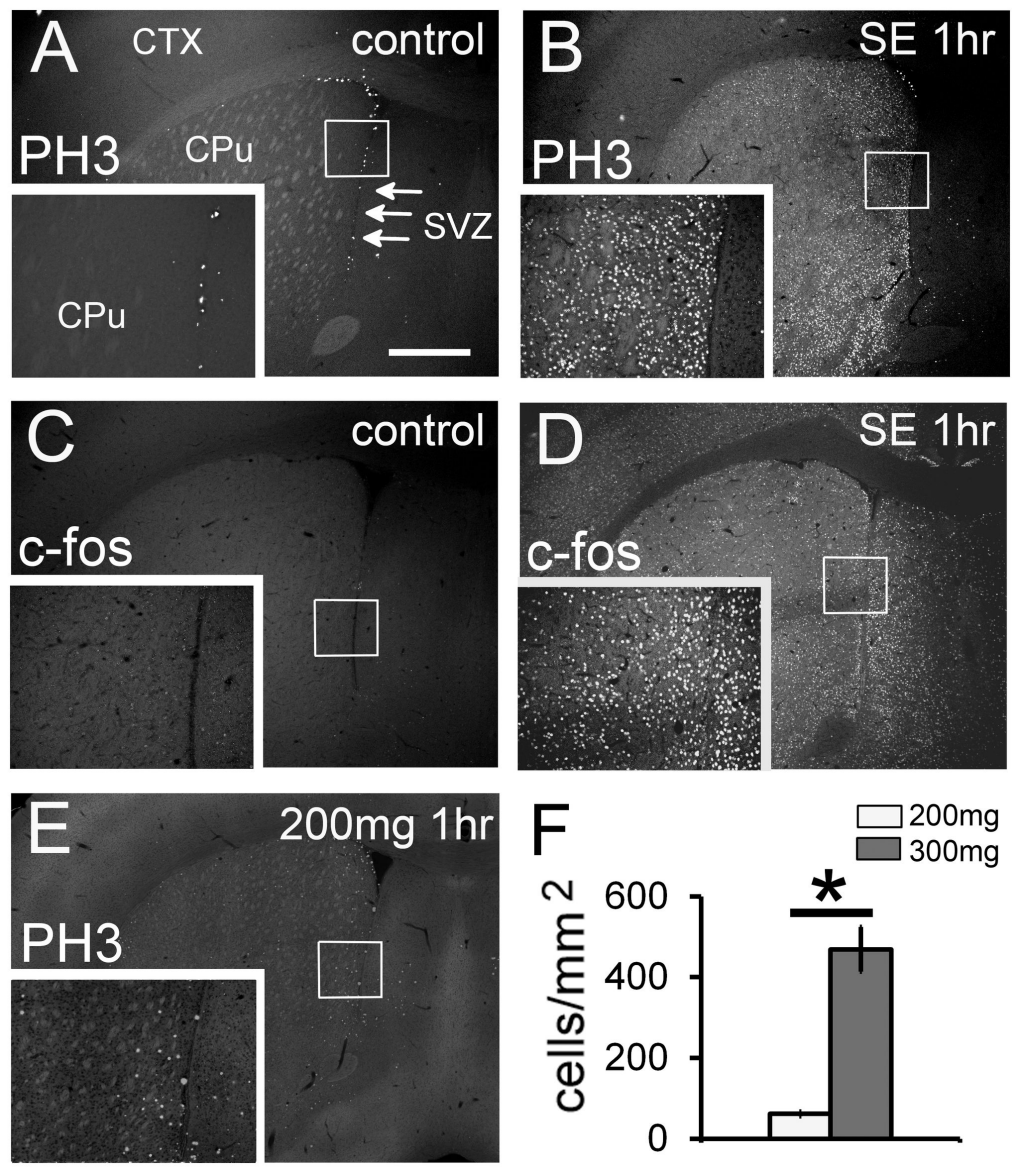
H3 phosphorylation is upregulated in the SE brain. Stacked epifluorescence microscopy images are shown. Images of the control (A and C) and status epilepticus brain at 1 h (SE 1 h, B and D) showing PH3^+^ (A and B) and c-fos^+^ (C and D) cells. Note that strong PH3 signals are detected in the SVZ (arrows) in the control group. Proliferating neural precursors are PH3^+^. (A) In the control brain, there are virtually no PH3^+^ cells in the parenchyma. (B) In the CPu of the SE group (300 mg/kg of pilocarpine), there are many PH3^+^ cells. There is a small population of c-fos^+^ cells in the control brain (C). In contrast, numerous c-fos^+^ cells are present in the SE brain (D). (E and F) At a subconvulsive dose of pilocarpine (200 mg/kg), the density of PH3^+^ cells is significantly low. Three animals in each group were analyzed (*n* = 3). Two-tailed Welch’s *t*-test. *p < 0.05. White bar, 200 mg/kg group; gray bar, 300 mg/kg group. Insets are higher magnification images of the boxed areas. CPu: caudate putamen; CTX: cortex; SVZ: subventricular zone. Scale bar = 600 μm for low magnification images and 300 μm for high magnification images.

There were virtually no PH3^+^ cells in the control brain parenchyma, except in the SVZ (Figure 1A). In fact, PH3 is also used as a marker of the G2/M phase of the cell cycle [22], and there are many proliferating neuronal stem/precursor cells in the adult SVZ [23]. In comparison, a prominent PH3 signal was detected in the CPu and Acb 1 h after SE induction, while the lateral septum was nearly free of PH3 immunoreactivity (Figure 1B). There are many cells expressing NG2, a chondroitin sulfate proteoglycan, in the normal brain parenchyma [24]. A few NG2^+^ cells can be found proliferating in the normal brain, but excessive neuronal excitation enhances entry into the cell cycle [25]. To exclude the possibility that NG2^+^ cells begin to proliferate after SE, we performed immunohistochemistry with a cell cycle marker, Ki67. Ki67 is expressed throughout the cell cycle, with the highest expression in the late G1 and S phase. Ki67^+^ cells were detected only in the SVZ, and not in the brain parenchyma of SE mice (Figure S1).

Many studies suggest that chromatin remodeling, an epigenetic mechanism of gene control, regulates gene expression [5]. A well-known target gene is c-fos [10]. At 1 h, c-fos^+^ cells were distributed all over the brain in SE mice (Figure 1D), but not in control mice (Figure 1C). It is notable that PH3^+^ cells were localized to specific brain structures (Figure 1B and see below). These signals obtained with the anti-PH3 antibody were specific for phosphorylated H3, because the PH3 signal disappeared after antibody absorption with blocking peptide (data not shown).

### H3 phosphorylation depends on neuronal excitation level

The severity of seizure behavior depends on the dose of pilocarpine, and behavioral phenomena correlate well with electroencephalogram (EEG) changes [26]. At a subconvulsive dose of 200 mg/kg, mice exhibited only repeated head-nodding movement. At a convulsive dose of 300 mg/kg, mice exhibited periodical generalized clonic seizures and continuous wed-dog shakes for several hours. The number of PH3^+^ cells in the CPu and Acb was significantly higher in the 300 mg/kg group compared with the 200 mg/kg group (Figure 1E and F). It is notable that, in the 200 mg/kg group, majority of PH3^+^ cells localized in the most medial side of the CPu (Figure 1E). Next, the distribution of c-fos^+^ cells in the 200mg/kg group was examined. Unlike in the 300 mg/kg group (Figure 1D), c-fos signals were detected in the restricted structures: for example, Cg, BNST, piriform cortex (Pir) (Figure S2). It is notable that in the CPu, c-fos^+^ cells localized in the medial CPu (Figures S2B and S2C). 

Administration of diazepam, an agonist of GABA-A receptors, can halt seizure. To examine the effect of seizure cessation, mice received diazepam 1 h after pilocarpine administration and were analyzed 1 h later. The number of PH3^+^ cells decreased sharply in the CPu and Acb (Figure S3A). In contrast, the number of PH3^+^ cells was still higher 2 h after pilocarpine administration when mice did not receive diazepam (Figure S3B).

### Identification of PH3^+^ cells in the SE brain

**Figure 2:**
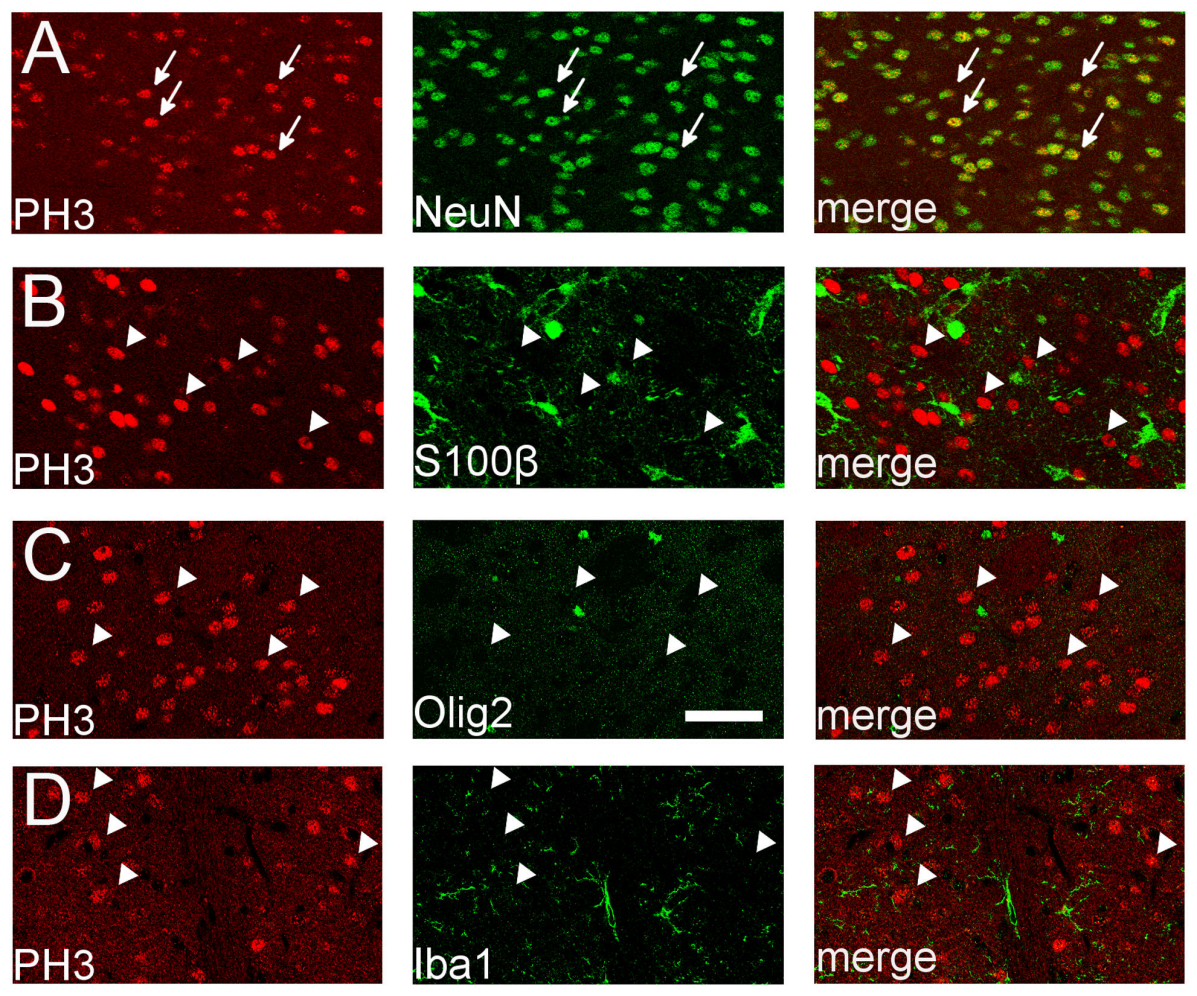
Identification of PH3^+^ cells in the CPu of the SE brain at 1 h. Identity of PH3^+^ cells (red) was determined by double immunostaining with cell type specific markers (green). Virtually all the PH3^+^ cells are NeuN^+^ mature neurons (A), not S100β^+^ astrocytes (B), Olig2^+^ oligodendrocytes/oligodendrocyte precursors (C) or Iba1^+^ microglia (D). Arrows indicate PH3^+^/NeuN^+^ (double-labeled) cells and arrowheads indicate PH3^+^ (single-labeled) cells. Single optical confocal microscopy images are shown. Scale bar = 50 μm.

To determine the identity of the PH3^+^ cells in the SE brain, we performed double immunostaining with PH3 and a cell type specific marker. Virtually all the PH3^+^ cells in the CPu and Acb 1 h after SE were NeuN^+^ mature neurons (Figure 2A). None of these PH3^+^ cells were S100β^+^ astrocytes (Figure 2B), Olig2^+^ oligodendrocytes/oligodendrocyte precursors (Figure 2C) or Iba1^+^ microglia (Figure 2D). Outside of the CPu, almost all the PH3^+^ cells were also NeuN^+^ neurons in the SE brain (see below).

### Distribution of PH3^+^ neurons after SE

**Figure 3:**
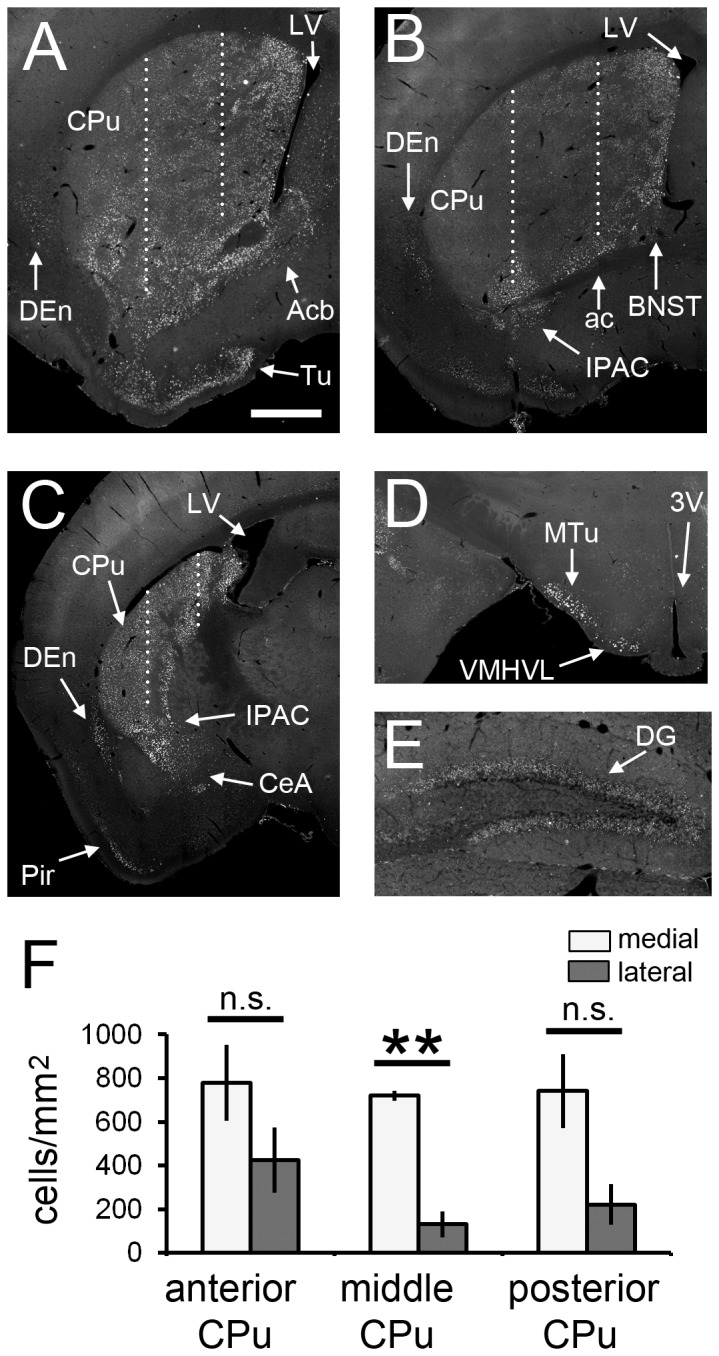
Distribution of PH3^+^ cells in the SE brain at 1 h. Anterior (A), middle (B) and posterior (C) parts are shown (see Materials and Methods). Stacked epifluorescence microscopy images are shown. Significant signals are detected in the CPu (A-C), nucleus accumbens (A), bed nucleus of the stria terminalis (B), interstitial nucleus of the posterior limb of the anterior commissure (B and C), dorsal endopiriform nucleus (A-C), piriform cortex (C), central amygdala (C), medial tuberal nucleus (D), ventromedial hypothalamic nucleus, ventrolateral part (D) and dentate gyrus of the hippocampus (E). (F) The CPu was divided into three parts with vertical lines (dotted lines in A-C; see Materials and Methods for detail), and the densities of PH3^+^ cells were compared between the medial and the lateral regions. Three animals were analyzed (*n* = 3). Two-tailed Welch’s *t*-test. **p < 0.01. White bars, medial region of CPu; gray bars, lateral regions of CPu; n.s., not significant. 3V: third ventricle; ac: anterior commissure; Acb: nucleus accumbens; BNST: bed nucleus of the stria terminalis; CeA: central amygdala; DEn: dorsal endopiriform nucleus; dG: dentate gyrus of the hippocampus; IPAC: interstitial nucleus of the posterior limb of the anterior commissure; LV: lateral ventricle; MTu: medial tuberal nucleus; Pir: piriform cortex; Tu: olfactory tubercle; VMHVL: ventromedial hypothalamic nucleus, ventrolateral part. Scale bar = 600 μm for A-D and 200 μm for E.

We analyzed the distribution pattern of PH3^+^ neurons 1 h after SE. As described above, prominent PH3 signals were detected in the CPu and Acb, as well as in the following specific structures: olfactory tubercle, BNST, IPAC, dorsal endopiriform nucleus (DEn), Pir, central amygdala (CeA), medial tuberal nucleus (MTu), ventromedial hypothalamic nucleus, ventrolateral part (VMHVL), DG and amydgalohippocampal area (Figure 3A-E, Figure S4). Anterior part of the pyramidal cell layer of the hippocampus was almost free of PH3 signal, but there were many PH3^+^ cells in the posterior part (Figures S4B and S4C). In general, the more posterior part of the brain was almost free of PH3 signal excepting the cortical area (Figures S4C and S4D). Interestingly, when we divided the CPu into three parts—medial, middle and lateral using equidistant vertical lines through the anterior, middle and posterior CPu—and compared the density of PH3^+^ neurons between the medial and the lateral part, there was a gradient along the medio-lateral axis. In the middle CPu, there were more PH3^+^ neurons in the medial part (Figure 3B and 3F). Although there was no significant difference in the posterior CPu, there was a trend towards a density gradient (*p* = 0.063, *n* = 3, Welch’s *t*-test; Figure 3C and 3F). In the anterior CPu, the density gradient was not obvious (Figure 3A and 3F).

In the cerebral cortex, there were virtually no PH3^+^ cells in the control brain (Figure S5A, S5C and S5E). In the SE brain, an intense signal was detected in the PL and the upper layer of the Cg (Figures S5D and S6B), while a moderate signal was detected in the upper (layers 2 and 3) and lower (layer 6) layers of the SM (Figure S5F). In contrast, the middle layer of the SM was almost free of PH3 signal (Figure S5F). The density of PH3^+^ cells was significantly higher in the PL (Figure S6C). Although there was no statistical significance between the Cg and SM, there was a trend towards higher density of PH3^+^ cells in the Cg than in the SM (Figure S6C). The PH3 signal in these regions was dramatically decreased in the mice subjected to SE cessation with diazepam administration (data not shown).

Strong neuronal excitation induces neuronal degeneration. Damaged neurons can be detected by FJC staining, and detailed time-course of FJC^+^ neurons was shown in the similar experimental condition to the present study [4]. To confirm neuronal damage in our experimental condition, we performed FJC staining at 4 and 8 h after pilocarpine administration. In general, FJC^+^ cells were diffusely detected and increased with time (data not shown). At 8 h, many FJC^+^ neurons were already detected in the amygdaloid complex (Figures S7B and S7C). These results are consistent with the literature [4]. It is notable that distribution of FJC^+^ cells was much broader than that of PH3^+^ cells (compare Figure 3C, S4A, S7B and S7C) and substantial number of PH3^+^ cells was detected in the CeA 1 h after administration of a subconvulsive dose of pilocarpine (200 mg/kg) (Figure S7D). 

### H3 phosphorylation in neurons in another SE model

**Figure 4:**
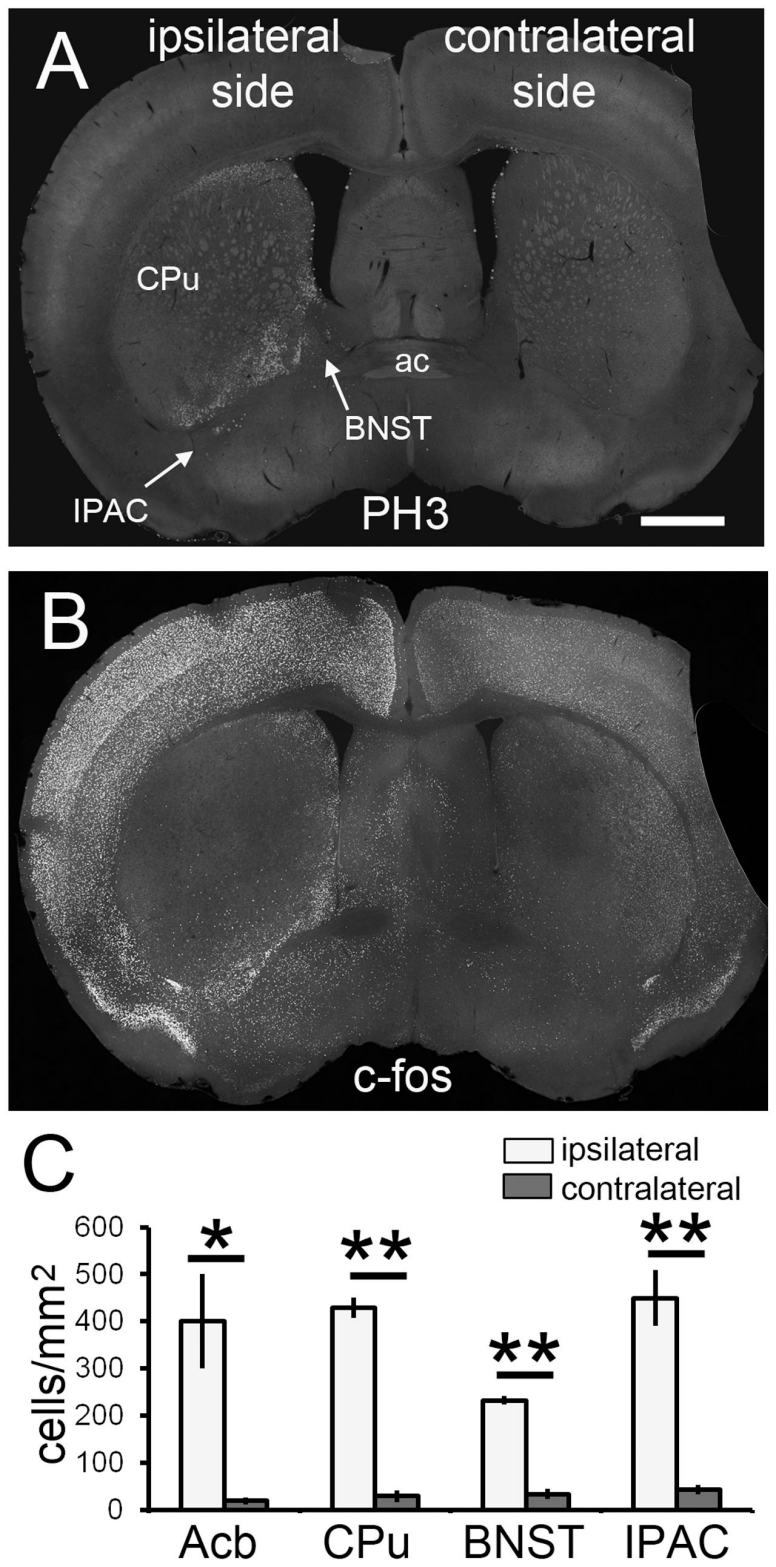
H3 phosphorylation level is also upregulated in specific brain structures of the kainic acid (KA)-induced SE brain. (A) H3 phosphorylation is greatly increased in the ipsilateral side in the KA-induced SE brain. (B) c-fos expression is also greatly upregulated in the ipsilateral side. Note that the distribution of c-fos^+^ neurons is much broader than that of PH3^+^ neurons, especially in the cerebral cortex. Stacked epifluorescence microscopy images are shown. Scale bar = 600 μm. (C) Densities of PH3^+^ neurons are significantly higher in the ipsilateral side compared with the contralateral side. Four animals were analyzed (*n* = 4). Two-tailed Welch’s *t*-test. *p < 0.05; **p < 0.01. White bars, ipsilateral side; gray bars, contralateral side.

Pilocarpine acts as an agonist of muscarinic acetylcholine receptors. There is a possibility that H3 phosphorylation in neurons is a specific effect of pilocarpine-induced SE. To exclude this possibility, we examined H3 phosphorylation in another SE model, KA-induced SE. KA solution was unilaterally injected into the basolateral amygdala to induce SE, and we then examined H3 phosphorylation. H3 phosphorylation was significantly induced in the ipsilateral hemisphere (Figure 4A and 4C). c-fos expression was also dramatically increased, especially in the ipsilateral hemisphere (Figure 4B). Again, c-fos^+^ neurons were more broadly distributed than PH3^+^ neurons in the KA-induced SE brain, similar to the pilocarpine-induced SE brain.

### Mechanisms of H3 phosphorylation

Studies suggest that H3 phosphorylation is mediated through the NMDA receptors/MAPK/MSK1/2 (mitogen and stress-activated kinase) pathway under stressful conditions [14,27,28]. First, we examined the role of NMDA receptors. MK-801, an antagonist of NMDA receptors, has an anticonvulsive effect [29], but administration of MK-801 at a dose of 4 mg/kg 20 min prior to pilocarpine does not block the onset of SE as determined by EEG analysis [30]. When MK-801 was administrated immediately after the first attack of generalized clonic seizures, the number of PH3^+^ cells decreased sharply in the CPu and Acb, but non-negligible number of PH3^+^ cells were still detected in the other structures including BNST, CeA, MTu and VMHVL (Figure S8). Similar result was obtained when MK-801 was administrated 20min prior to pilocarpine (data not shown). 

**Figure 5:**
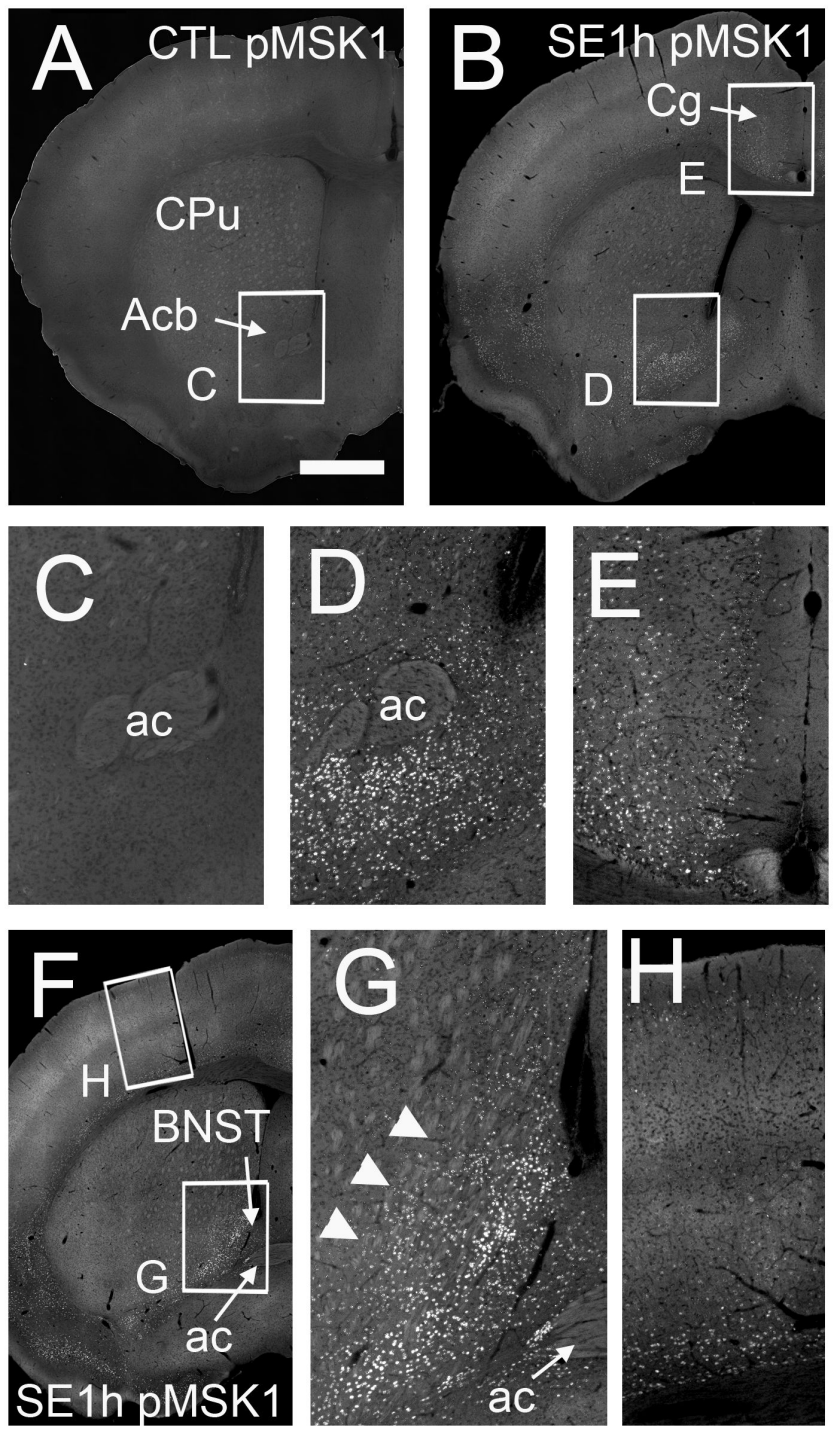
MSK1 is phosphorylated in the pilocarpine-induced SE brain at 1 h. Stacked epifluorescence microscopy images of the anterior (A-E) and middle (F-H) part of the brain of the control (A and C) and SE 1 h group (B and D-H) are shown. High magnification images of the Acb (C and D), Cg (E), BNST (G), ventral limb of the CPu (arrowheads, G), and the somatosensory cortex (H) are shown. (A and C) In the control brain, there are virtually no phosphorylated MSK1^+^ (pMSK1^+^) cells. The distribution of pMSK1^+^ cells is similar to that of PH3^+^ neurons in the SE brain at 1 h (see Figure 3 and Figure S5). Note that there are fewer pMSK1^+^ cells in the CPu (G). Cg: cingulate cortex. Scale bar = 600 μm for A, B and F, and 150 μm for C-E and G-H.

Second, we examined the distribution of phosphorylated MSK1^+^ (pMSK1^+^) cells in the SE brain. In the control mice, virtually no pMSK1 signal was detected (Figure 5A and 5C). One hour after injection of pilocarpine, phosphorylation of MSK1 was induced in the Acb, CPu, BNST, IPAC, Cg, and upper and lower layer of the cerebral cortex (Figure 5B and 5D-H). In the CPu, pMSK1 signals were localized close to the lateral ventricle and the ventral limb of the CPu (arrowheads in Figure 5G), and were very sparsely distributed in the core region (compare Figure 1B and Figure 5F and 5G). In the DG, the pMSK1 signal increased prominently (data not shown). Moreover, in the cerebral cortex, layers of pMSK1^+^ neurons were thinner than that of PH3^+^ neurons (compare Figure 5H and Figure S5F). Collectively, these results indicate that the distribution of pMSK1^+^ cells was similar to that of PH3^+^ cells, although the distribution of pMSK1^+^ cells was more restricted than that of PH3^+^ cells (Figure S9).

### H3 phosphorylation in the CPu after ablation of dopaminergic neurons

The CPu is a component of the basal ganglia and plays important roles in motor control [31]. Recently, experimental data suggest that the basal ganglia does not trigger seizures, but propagate and control seizures through the cortico-basal ganglia-cortical circuit [32]. Thus, we decided to examine further the regulation of H3 phosphorylation in the CPu neurons. 

It has been reported that combined activation of dopamine D1 receptor and NMDA receptors increases H3 phosphorylation in CPu neurons [18-21]. Because axon terminals of dopaminergic neurons projecting ipsilaterally from the substantia nigra are dense in the CPu and Acb, we hypothesized that H3 phosphorylation in the CPu is regulated through the activation of dopamine receptors. To examine this possibility, dopaminergic neurons in the substantia nigra were ablated by injecting 6-OHDA unilaterally into the medial forebrain bundle.

**Figure 6:**
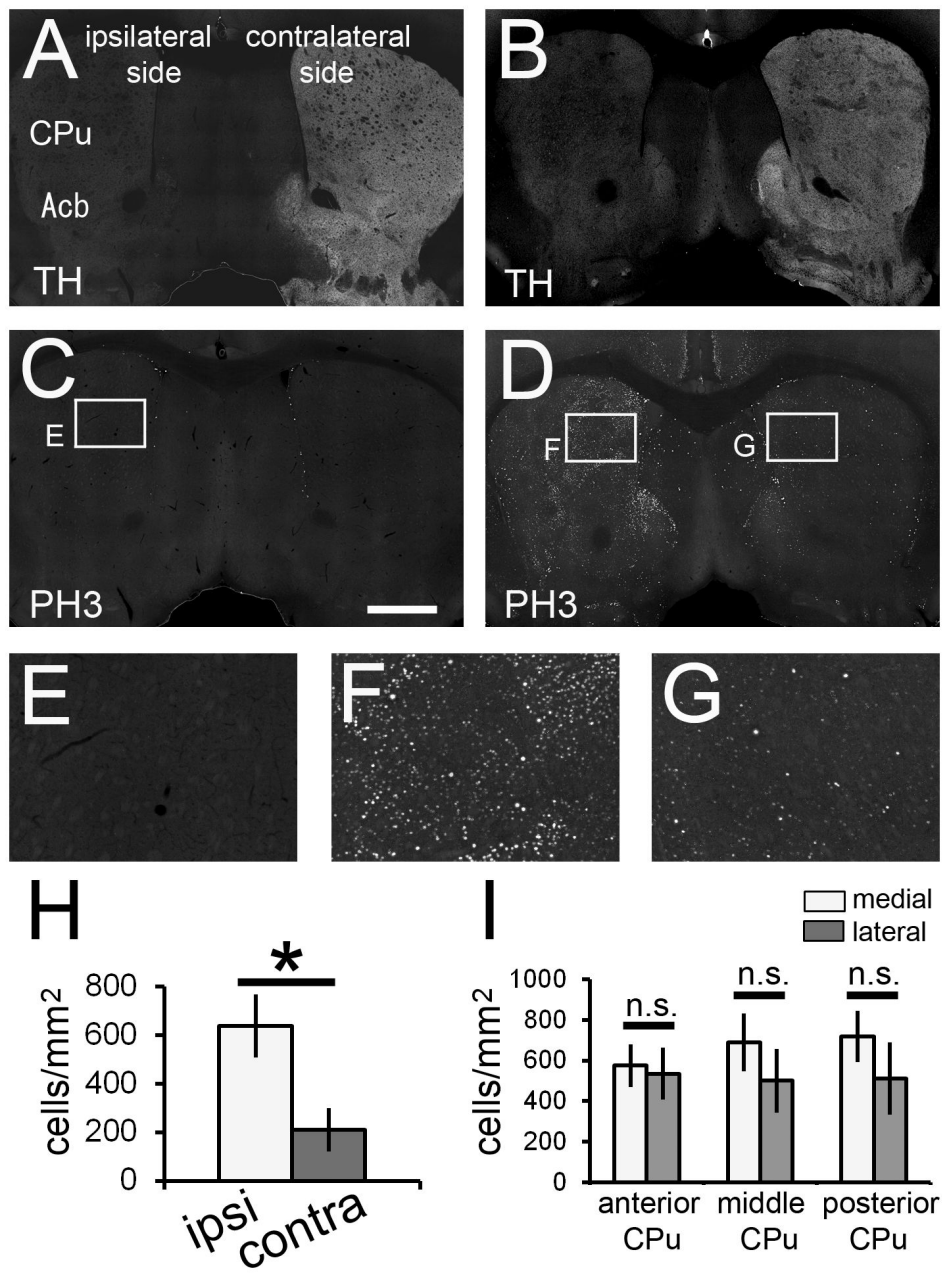
Dopaminergic input from the substantia nigra affects H3 phosphorylation in the CPu of the pilocarpine-induced SE brain. Unilaterally 6-hydroxydopamine (6-OHDA)-injected mice were subjected to SE induction. Double immunostaining with anti-tyrosine hydroxylase (TH) and anti-PH3 antibodies was performed on the vehicle-administered control brain section (A, C and E) and the pilocarpine-induced SE brain section (B, D, F and G). TH immunoreactivity in the CPu and Acb is reduced significantly in the 6-OHDA injection side (A and B). H3 phosphorylation is significantly increased in the SE brain, especially in the ipsilateral side (D, F and G). Stacked epifluorescence microscopy images are shown. High magnification images of the boxed areas are shown in each image. (H) Density of PH3^+^ neurons in the CPu is significantly higher in the ipsilateral side than in the contralateral side of the pilocarpine-induced SE brain. White bar, ipsilateral side; gray bar, contralateral side. (I) Density gradient of PH3^+^ neuron in the CPu is lost in the 6-OHDA-injected side. White bar, medial part of the CPu; gray bar, lateral part of the CPu. Five animals were analyzed (*n* = 5). Two-tailed Welch’s *t*-test. *p < 0.05. Scale bar = 600 μm for A-D and 150 μm for E-G.

**Figure 7:**
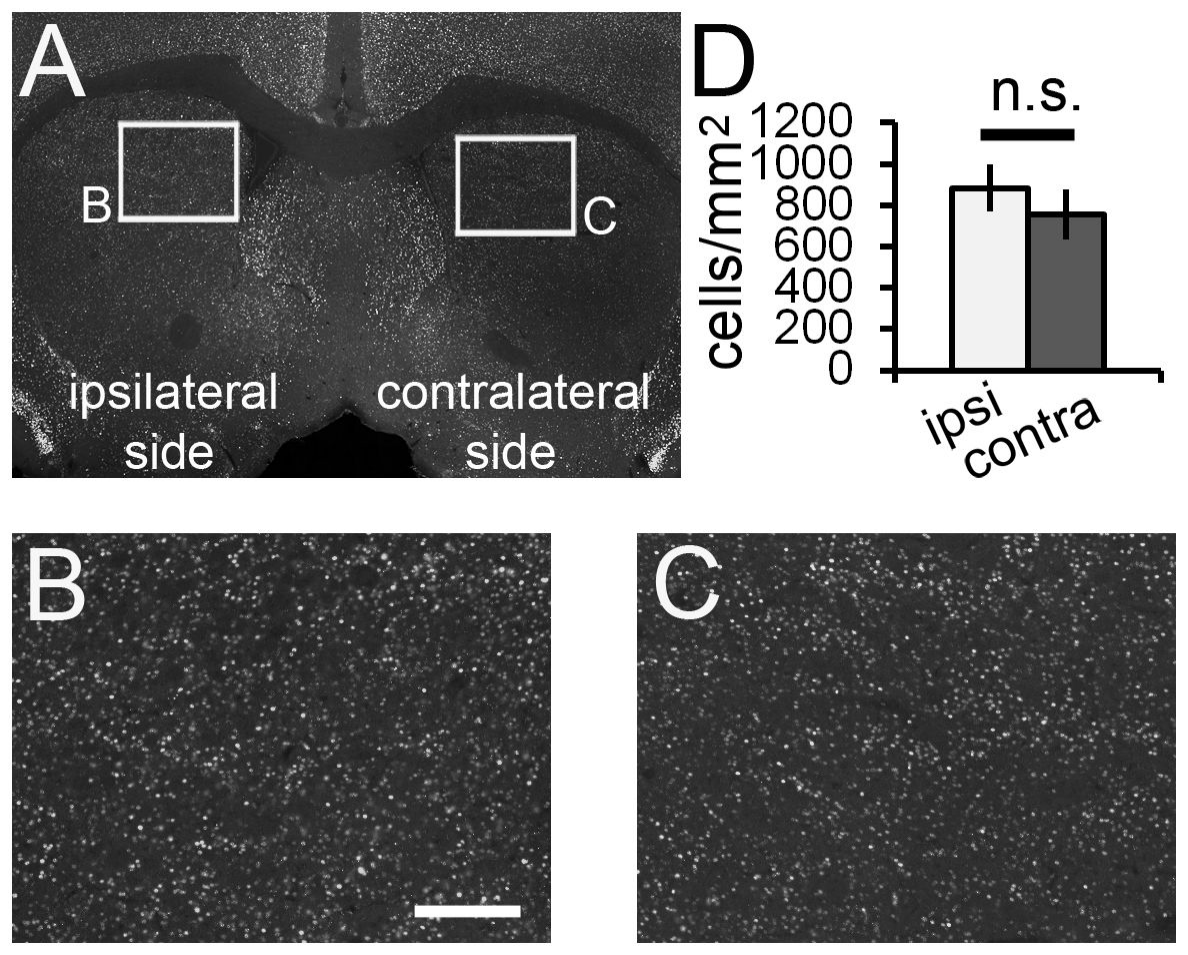
c-fos expression is not affected by ablation of dopaminergic input to the CPu. Stacked epifluorescence microscopy images are shown. High magnification images of the boxed areas in (A) are shown (B and C). There is no significant difference in the density of c-fos^+^ cells between the ipsilateral and contralateral sides after unilateral 6-OHDA injection and SE induction with pilocarpine (D). White bar, ipsilateral side; gray bar, contralateral side. Five animals were analyzed (*n* = 5). Two-tailed Welch’s *t*-test. Scale bar = 600 μm for A and 150 μm for B and C.

Three weeks after 6-OHDA injection, immunoreactivity for TH, a critical enzyme in the production of catecholamines, was greatly decreased in the ipsilateral side of the CPu (Figure 6A and 6B). Subsequently, these mice were used for the SE experiment. In the control mice receiving 6-OHDA, scopolamine and PBS, there were virtually no PH3^+^ cells in the brain parenchyma, apart from the SVZ (Figure 6C and 6E). In the experimental mice receiving 6-OHDA, scopolamine and pilocarpine, 1 h after pilocarpine injection, H3 phosphorylation was significantly increased in the CPu in the ipsilateral hemisphere (Figure 6D and 6F-H). Furthermore, the density gradient of the PH3^+^ neurons in the CPu described above (Figure 3F) disappeared after ablation of dopaminergic neurons in the ipsilateral side (Figure 6D and 6I). Interestingly, there was no significant difference in the density of c-fos^+^ neurons between the ipsilateral and contralateral sides of the CPu (Figure 7).

## Discussion

Although many studies have demonstrated that several stimuli, such as stress and seizures, increase H3 phosphorylation in hippocampal neurons, studies of other regions are limited. In this study, we demonstrate that SE induces H3 phosphorylation in neurons in specific brain structures.

### H3 phosphorylation level depends on neuronal excitation

Administration of pilocarpine at a subconvulsive dose induced much fewer PH3^+^ neurons than at the convulsive dose. Moreover, the number of PH3^+^ neurons dramatically decreased after SE cessation by diazepam administration. Because behavioral phenomena correlate with EEG changes [26], our results suggest that H3 phosphorylation in neurons greatly depends on neuronal excitation. 

In the pilocarpine-induced SE brain, a prominent increase in H3 phosphorylation was detected in neurons in a limited number of brain structures, including the olfactory tubercle, Acb, CPu, BNST, IPAC, CeA, DEn, Cg, Pir, MTu, VMHVL and DG. In particular, in the CPu, there was a density gradient of PH3^+^ neurons along the medio-lateral axis. 

c-fos^+^ neurons distributed in almost all regions in the SE brain. In contrast, at a subconvulsive dose of pilocarpine, c-fos^+^ cell distributed in more limited structures, including the Cg, BNST and Pir. In particular, in the CPu, c-fos^+^ cells localized in the medial part, and lateral part was almost free of PH3 signal. These results suggest that neurons in these structures tend to be excited after induction of seizures. These structures correspond to PH3 signal-dense structures in the SE brain. The CPu receives input from the cortex with a topographical organization pattern [33]. The medial part of the CPu receives inputs from the PL and the Cg, and these cortical regions contain many PH3^+^ neurons in the SE brain. In comparison, the lateral part of the CPu receives input from the SM, and this cortical region contains less PH3^+^ neurons in the SE brain. These results suggest that there are more PH3^+^ neurons in the strongly excited regions compared with the weakly excited regions.

c-fos upregulation is frequently used as a marker of neuronal excitation. But c-fos might be too sensitive an indicator of the response to various stimuli. Our present results suggest that PH3 is a suitable marker of strong neuronal excitation.

### SE induces H3 phosphorylation in neurons in specific brain structures

Pilocarpine acts as an agonist of muscarinic acetylcholine receptors, and cholinergic terminals are dense in the Acb, CPu and Tu, as revealed by acetylcholine esterase staining [34]. However, upregulation of H3 phosphorylation is not specific to the pilocarpine-induced SE brain, because a similar phenomenon is observed in the ipsilateral side of the unilaterally KA-injected brain. In fact, in an *in vitro* culture system of striatal neurons, neuronal excitation mediated by activation of the glutamate receptors/MAPK/MSK1 pathway induces H3 phosphorylation [16]. These data indicate that H3 phosphorylation in neurons outside the hippocampus is a general phenomenon in the SE brain.

Why were the PH3^+^ neurons localized to specific brain structures? Are there any functional relationships or neural connections between these brain structures? The CeA and medial amygdala, BNST, sublenticular extended amygdala and IPAC are strongly interconnected and compose the extended amygdala, which is in turn divided into two parts, the central and medial extended amygdala [35,36]. In particular, the CeA, the lateral division of the BNST, the IPAC and the central division of the sublenticular extended amygdala form the central division of the extended amygdala and establish reciprocal connections with the caudal aspect of the Acb shell and with the prefrontal cortex [36]. Clinical studies show that human patients with TLE have psychiatric disorders, such as mood disorders (49.3%), depression (27.4%), anxiety disorders (42.5%) and bipolar disorder (9.6%) [37]. In fact, the extended amygdala is involved in fear and anxiety responses [38,39], and TLE patients with ictal fear have atrophy of the amygdala [40]. Although high dose of pilocarpine in the experimental animals induces neuronal damage in the amygdaloid complex as shown in the present study, the relation between pilocarpine model of TLE and fear/anxiety response is controversial: high dose of pilocarpine reduces anxiety-like behavior and impair fear conditioning [41], increases anxiety-like behavior [42] or impairs extinction of fear memory with no differences in basic anxiety levels [43]. These conflicting results might depend on the severity of neuronal damage due to differences in experimental conditions, such as animal species (rat or mouse), strains of animals and doses of pilocarpine. In the present study, strong PH3^+^ neurons localized specifically in the CeA, but damaged neurons were diffusely detected in the amygdaloid complex including the CeA after SE induction. This result suggests that H3 phosphorylation can regulate some other genes with the exception of cell death-related genes, and those genes can modulate neuronal excitation and/or re-organize neural circuit in the amygdaloid complex leading to fear/anxiety responses as late onset effects of pilocarpine administration. Further study is needed to clear this issue. 

### Dopaminergic system modulates H3 phosphorylation in the SE brain

The cortico-basal ganglia-cortical loop is a well-known circuit that plays a crucial role in the functioning of the basal ganglia. Glutamate and dopamine are key neurotransmitters in this system [31]. Recently, the relationship between the dopamine system and the pathophysiology of epilepsy has been attracting increasing attention [44].

Dopamine receptors are G-protein-coupled trans-membrane receptors, and can be divided into two subtypes; D1-like (D1 and D5) and D2-like (D2, D3, and D4). Medium spiny neurons (MSNs) expressing NMDA receptors are projection neurons representing about 95% of striatal neurons. MSNs expressing D1 receptor in the striatum project to the internal segment of the globus pallidus and the substantia nigra reticulata, while MSNs expressing D2 receptor project to the external segment of the globus pallidus [45].

Studies have shown that combined activation of D1 receptor and NMDA receptors results in H3 phosphorylation in MSNs [18,19,21] and dopamine release is increased in the SE brain [46]. Therefore, we conjectured that if dopaminergic neurons in the substantia nigra projecting to the CPu were ablated, H3 phosphorylation should decrease. We unexpectedly found that unilateral depletion of dopaminergic neurons decreased H3 phosphorylation, not in the ipsilateral side of the CPu, but in the contralateral side, even though there was no significant difference in c-fos expression between the two hemispheres. Present study showed that inactivating NMDA receptors by MK-801 decreased sharply the number of PH3^+^ cells at least in the CPu. Thus, glutamatargic afferents from the cortex are critical for induction of H3 phosphorylation in the CPu of the SE brain. MSNs receive two types of glutamatergic input from the cortex: (i) intratelencephalic (IT) neurons projecting to both the ipsilateral and contralateral striatum, and (ii) pyramidal tract (PT) neurons ipsilaterally projecting to the pyramidal tract with collateral projections to the striatum. Input from IT neurons is the major input for both populations of MSNs, and ablation of dopaminergic innervation specifically inhibits activity of IT neurons [47]. Glutamate and dopamine D1 receptor signaling pathways converge, resulting in activation of MAPK/ERK. Activation of glutamate receptors induces H3 phosphorylation in cultured striatal neurons (discussed further below) [16,48]. Thus, in the unilaterally 6-OHDA-injected animals, the number of PH3^+^ neurons in the contralateral CPu following SE might be diminished.

Importantly, the D2 receptor is also expressed at the presynaptic terminals of glutamatergic afferents from the cortex, and activation of D2-like receptors has an inhibitory effect on glutamate release [49]. In the ipsilateral CPu of unilaterally 6-OHDA-injected animals, even though activity of IT neurons is reduced, glutamate release might be enhanced by release from the inhibitory effect of the D2 receptor, and H3 phosphorylation might consequently be increased.

### Signaling pathways involved in H3 phosphorylation

Studies have shown that the MAPK/ERK/MSK1/2 signaling pathway interacting with NMDA receptors is critical for H3 phosphorylation induced by various stimuli in neurons [12,16,19,28,50]. In the present study, we found that the distribution of pMSK1^+^ neurons was similar to that of PH3^+^ neurons in the Acb, CPu, BNST, IPAC, Cg, upper and deep layer of the cortex, and the DG. This suggests that H3 phosphorylation in CPu neurons after SE might be directly linked to the activation of the MAPK/ERK/MSK1 pathway. However, it is notable that the distribution of pMSK1^+^ neurons was much more limited than that of PH3^+^ neurons, especially in the CPu. In various types of cells, H3 phosphorylation is mediated by other kinases, such as RSK2, Cot, Aurora kinases, IKK-alpha and PIM1 [51-55]. Therefore, it is possible that some of these kinases participate in H3 phosphorylation through other signaling pathways in the SE brain.

What are the target genes of histone modification in the SE brain? A well-known target of H3 phosphorylation is c-fos, suggested by direct and indirect evidences from various experimental paradigms [11,12,14]. Our results show that the distribution of c-fos^+^ cells is consistently larger than that of PH3^+^ cells, such as in the lateral part of the middle CPu. A similar phenomenon is observed in the amphetamine-administered brain [56]. These findings suggest that, in addition to H3 phosphorylation, there are additional mechanisms mediating c-fos induction. This hypothesis is supported by the observations of Bilang-Bleuel et al (2005). They showed that there is a large temporal gap between H3 phosphorylation and c-fos expression in the hippocampus in the forced swimming experimental paradigm [27]. However, it is possible that a very low level of H3 phosphorylation, below the sensitivity limit of the immunohistochemical method, might be sufficient to induce transcription of the c-fos gene.

Other candidate genes regulated by H3 phosphorylation are the cell death-related genes. In the similar experimental condition to the present study, many damaged neurons in the CPu and other brain structures are detected by Fluoro-Jade C staining as early as 4 hours after administration of pilocarpine [4]. 

In summary, we show that H3 phosphorylation level in neurons is increased by excessive neuronal excitation. In the SE brain, prominent H3 phosphorylation was detected in a specific set of brain structures, and it was clearly affected by the dopamine-signaling pathway. The distribution of PH3^+^ neurons in the SE brain suggests functional neuronal connectivity among these brain structures. Furthermore, our findings suggest that H3 phosphorylation is a good indicator of strong neuronal excitation. Elucidating the molecular mechanisms mediating H3 phosphorylation and identifying the downstream target genes after induction of SE in the pilocarpine model of TLE should help advance our understanding of the pathogenetics of epilepsy. 

## Supporting Information

Figure S1 **There are virtually no proliferating cells in the brain parenchyma.** In both the control and SE 1 h brain, there are Ki67^+^ proliferating cells in the SVZ (arrows), but not in the brain parenchyma. Insets indicate higher magnification images of boxed areas in each image. Stacked epifluorescence microscopy images are shown. Scale bar = 600 μm for low magnification images and 300 μm for high magnification images.
(TIF)

Figure S2 **Subconvulsive dose of pilocarpine induces c-fos expression in the restricted structures.** Stacked pifluorescence microscopy images showing c-fos^+^ cells 1h after administration of a subconvulsive dose of pilocarpine (200 mg/kg). High magnification images of the medial (B) and lateral (C) part of the CPu, Cg (D) and BNST (E) are shown. Note that the density of c-fos^+^ cells in the CPu is much lower at 200 mg/kg than at 300 mg/kg (see Figure 1D), but a density gradient along the medio-lateral axis exists (B and C). Scale bar = 600 μm for A, 150μm for B-E.
(TIF)

Figure S3 **Sustained high H3 phosphorylation level without cessation of seizure.** Stacked epifluorescence microscopy images showing PH3^+^ cells in the CPu. Brains were taken 2 h (A, B) after pilocarpine administration with (A) or without (B) seizure cessation with diazepam administration 1 h before fixation. There are many PH3^+^ neurons in mice with prolonged seizures (B). Scale bar = 300 μm.
(TIF)

Figure S4 **PH3^+^ cells in the caudal region of the SE brain at 1 h.** Stacked epifluorescence microscopy images showing the amygdala and hippocampus (A-C), and the midbrain (D). Many PH3^+^ cells are detected in the CeA, but few in the basolateral amygdala (A). In the hippocampus, dense PH3 signal are detected in the DG and there are scattered PH3^+^ cells in the CA3 (B). In the more posterior part of the hippocampus, there are many PH3^+^ cells in the pyramidal cell layer (arrowheads) and the amydgalohippocampal area (C). The midbrain is free of PH3 signal (C and D). AHi: amydgalohippocampal area; Aq: aqueduct; BLA: basolateral amygdala; CA1: field CA1 of the hippocampus; CA2: field CA2 of the hippocampus; CA3: field CA3 of the hippocampus; Mam: mammillary body; MG: medial geniculate nucleus; SC: superior colliculus; SNR: substantia nigra, reticular part. Scale bar = 300 μm for A and D, 600 μm for B and C.
(TIF)

Figure S5 **PH3^+^ cells in the cortex of the SE brain at 1 h.** In the cortex of the control brain, there are virtually no PH3^+^ cells (A, C and E). In contrast, in the upper and lower layers of the neocortex and Cg, there are many PH3^+^ cells in the SE brain at 1 h (B, D and F). Stacked epifluorescence microscopy images showing the Cg (C and D) and primary motor cortex E and F). cc: corpus callosum. Scale bar = 600 μm for A and B, and 150 μm for C-F.
(TIF)

Figure S6 **The density of PH3+ cells is different between cortical areas.** Stacked epifluorescence microscopy images showing PH3^+^ cells in the prelimbic cortex 1h after SE induction (A and B). A higher magnification image of the boxed area in A is shown (B). (C) The density of PH3^+^ cells in the PL is significantly higher than the Cg and sensorimotor cortex. Three animals in each group were analyzed (*n* = 3). One-way ANOVA followed by Tukey’s post hoc test, * *p* < 0.05, ** *p* < 0.01. n.s., not significant. PL: prelimbic cortex; SM: sensorimotor cortex. Scale bar = 1200 μm for A and 300 μm for B.
(TIF)

Figure S7 **Damaged neurons are detected in the SE brain.** Stacked epifluorescence microscopy images showing Fluoro-Jade C (FJC)^+^ neurons 8 h after SE induction (A-C) and PH3^+^ cells 1 h after administration of a subconvulsive dose of pilocarpine (200 mg/kg) (D). (B and C) In the amygdaloid complex, many FJC^+^ neurons are detected, but there are relatively less FJC^+^ neurons in the BLA. Higher magnification images of the boxed areas in A are shown. (D) Substantial number of PH3^+^ cells was detected in the CeA at a subconvulsive dose of pilocarpine. BMA: basomedial amygdala; MeA: medial amygdala. Scale bar = 1200 μm for A, 300 μm for B, C and D.
(TIF)

Figure S8 **Effect of the NMDA receptor antagonist, MK-801.** MK-801 was administered immediately after the onset of generalized clonic seizures and analysis was performed 1h after MK-801. Stacked epifluorescence microscopy images showing PH3^+^ cells. High magnification images of the boxed areas are shown in each image. In the Acb and CPu, the number of PH3^+^ cells decreases dramatically (A, B, D, E and F). But there are still non-negligible number of PH3^+^ cells in the specific structures including the BNST, CeA, MTu and VMHVL (C and G-I). Scale bar = 1200 μm for A-C, 300 μm for D-I. .
(TIF)

Figure S9 **Distribution of pMSK1^+^ cells is similar to that of PH3^+^ cells.** (A-D) The distributions of PH3^+^ cells and pMSK1^+^ cells were compared by using serial sections. Stacked epifluorescence microscopy images are shown. The distribution of pMSK1^+^ cells is similar to that of PH3^+^ cells in the Acb, CPu and BNST, but the number of pMSK1^+^ cells is fewer than that of pH3^+^ cells (see also Figures 3, 5 and S5). (E and F) Single optical confocal microscopy images of the BNST are shown. Virtually all the NeuN^+^ mature neurons are PH3^+^ (arrows, E), but pMSK1^+^ neurons are a subpopulation (arrows, F). Arrows and arrwoheads indicate NeuN^+^/pMSK1^+^ (double labeled) cells and NeuN^+^/pMSK1^-^ (single labeled) cells respectively. Nuclei were stained in blue with Hoechst33258. Scale bar in D = 300 μm for A-D, scale bar in E = 50 μm for E and F.
(TIF)
