# Supplementary figures and images for: Increased Histone H3 Phosphorylation in Neurons in Specific Brain Structures after Induction of Status Epilepticus in Mice
Source: PLoS One. 2013 Oct 16;8(10):e77710. doi: 10.1371/journal.pone.0077710 (PMC3797699; doi:10.1371/journal.pone.0077710)

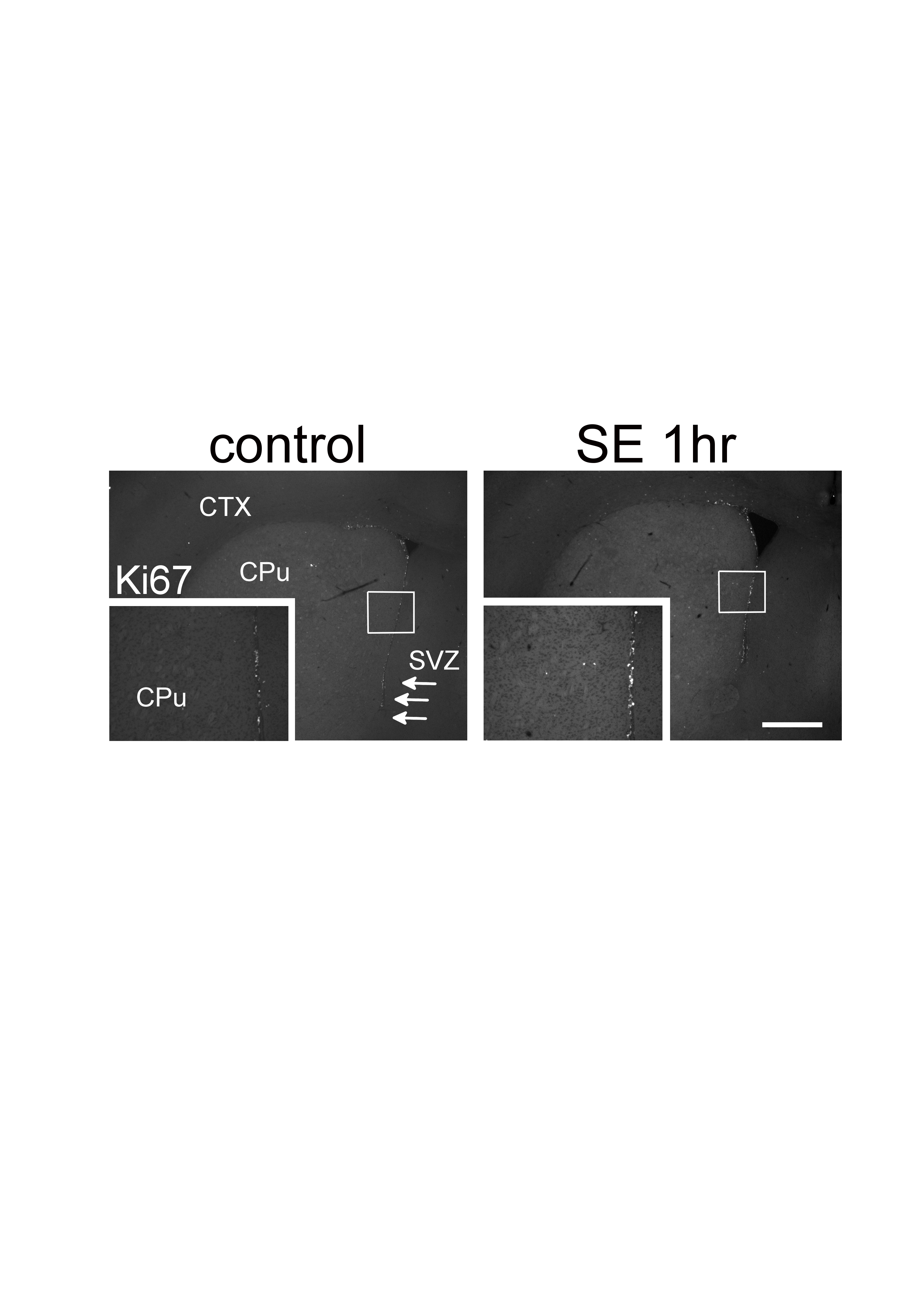

Supplement: Figure S1 — There are virtually no proliferating cells in the brain parenchyma. In both the control and SE 1 h brain, there are Ki67+ proliferating cells in the SVZ (arrows), but not in the brain parenchyma. Insets indicate higher magnification images of boxed areas in each image. Stacked epifluorescence microscopy images are shown. Scale bar = 600 μm for low magnification images and 300 μm for high magnification images. (TIF) [file pone.0077710.s001.tif]

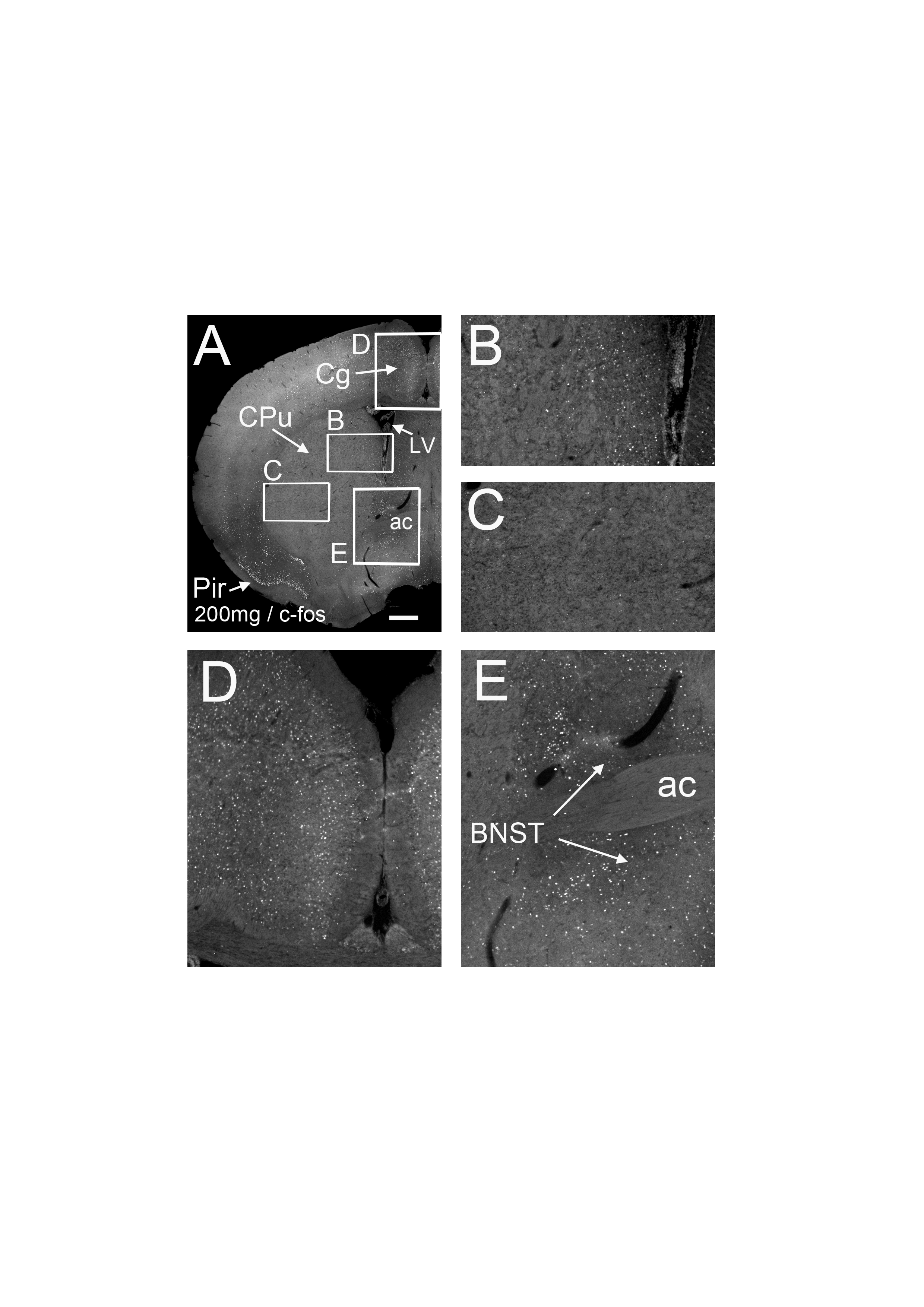

Supplement: Figure S2 — Subconvulsive dose of pilocarpine induces c-fos expression in the restricted structures. Stacked pifluorescence microscopy images showing c-fos+ cells 1h after administration of a subconvulsive dose of pilocarpine (200 mg/kg). High magnification images of the medial (B) and lateral (C) part of the CPu, Cg (D) and BNST (E) are shown. Note that the density of c-fos+ cells in the CPu is much lower at 200 mg/kg than at 300 mg/kg (see Figure 1D), but a density gradient along the medio-lateral axis exists (B and C). Scale bar = 600 μm for A, 150μm for B-E. (TIF) [file pone.0077710.s002.tif]

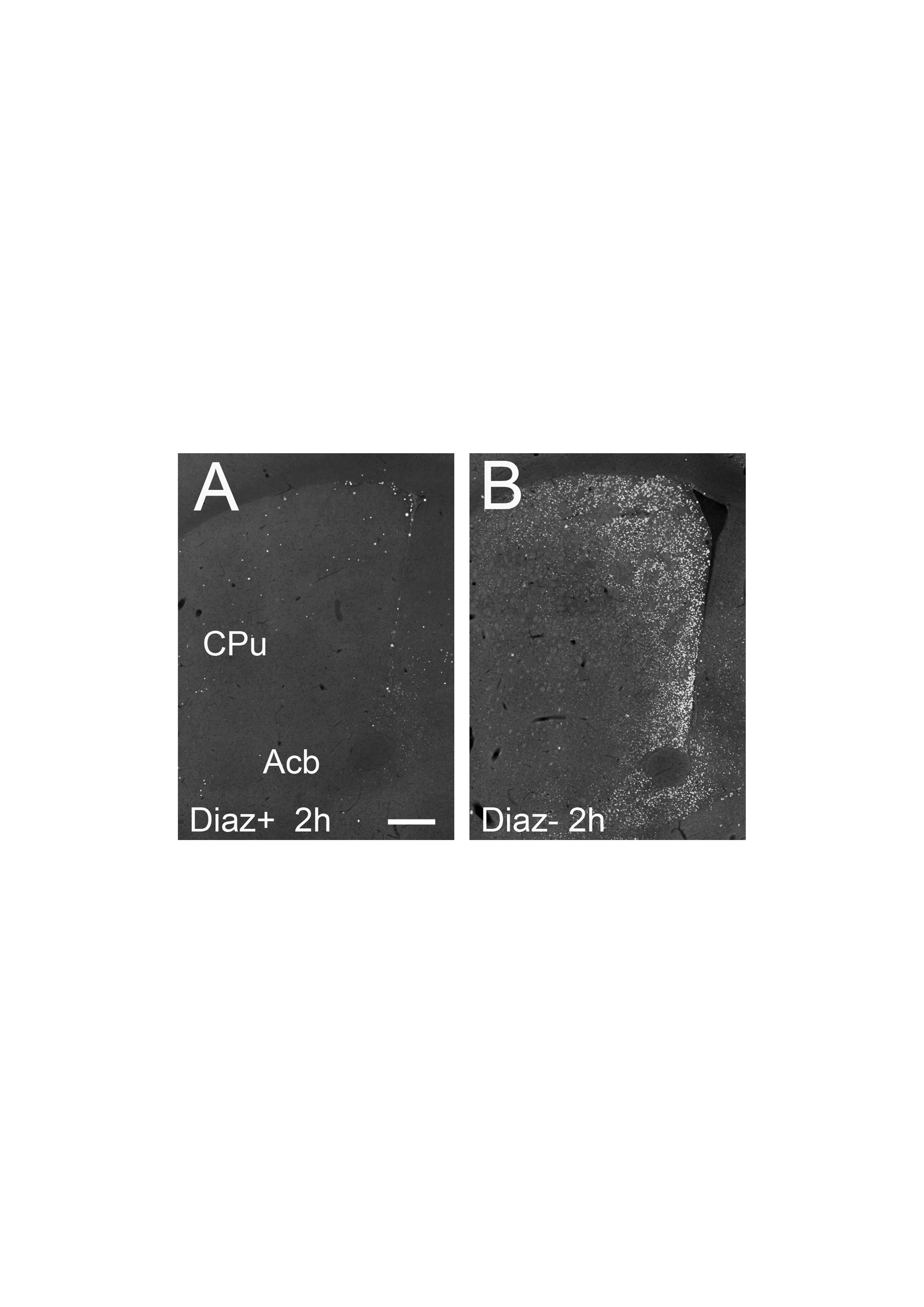

Supplement: Figure S3 — Sustained high H3 phosphorylation level without cessation of seizure. Stacked epifluorescence microscopy images showing PH3+ cells in the CPu. Brains were taken 2 h (A, B) after pilocarpine administration with (A) or without (B) seizure cessation with diazepam administration 1 h before fixation. There are many PH3+ neurons in mice with prolonged seizures (B). Scale bar = 300 μm. (TIF) [file pone.0077710.s003.tif]

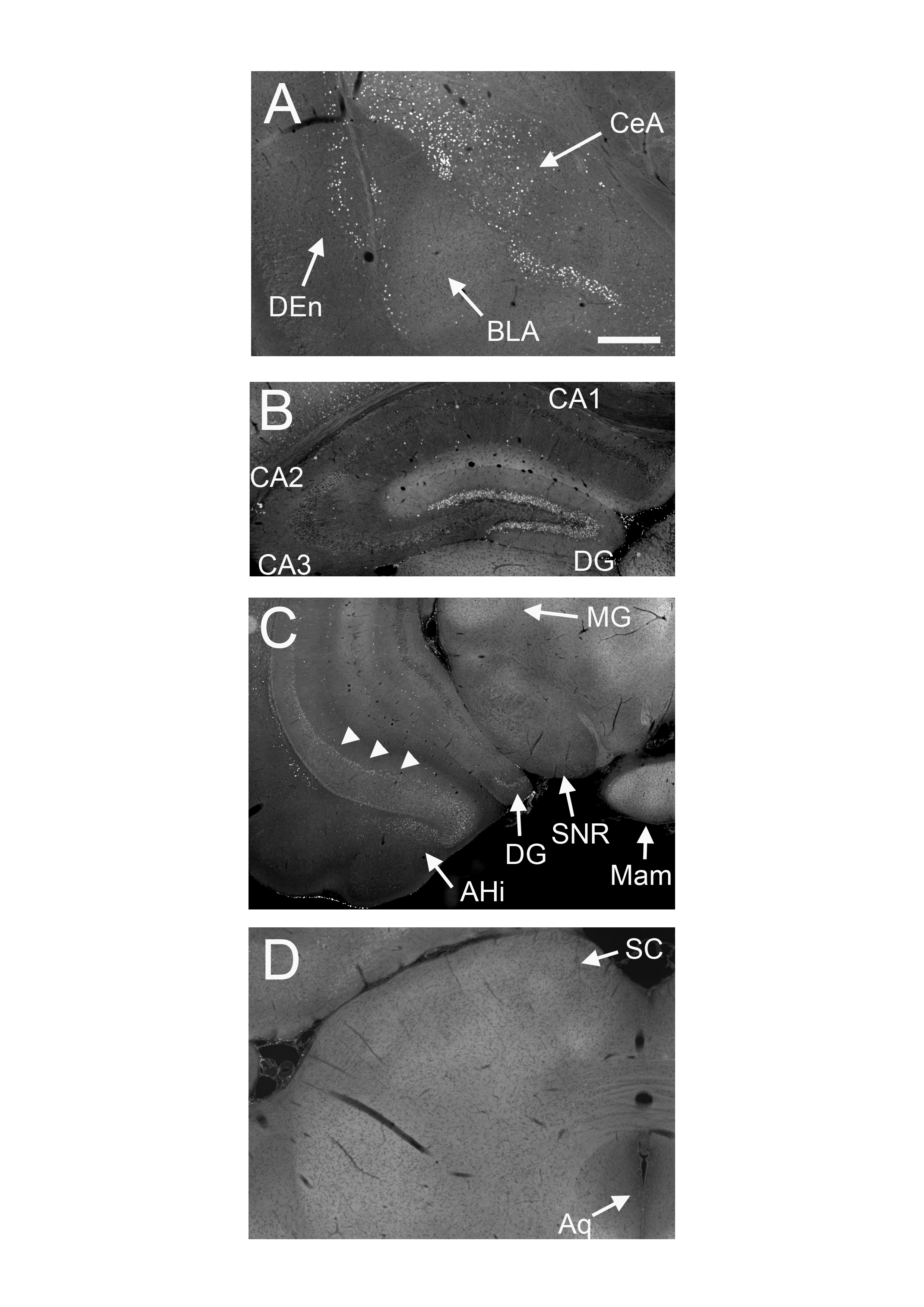

Supplement: Figure S4 — PH3+ cells in the caudal region of the SE brain at 1 h. Stacked epifluorescence microscopy images showing the amygdala and hippocampus (A-C), and the midbrain (D). Many PH3+ cells are detected in the CeA, but few in the basolateral amygdala (A). In the hippocampus, dense PH3 signal are detected in the DG and there are scattered PH3+ cells in the CA3 (B). In the more posterior part of the hippocampus, there are many PH3+ cells in the pyramidal cell layer (arrowheads) and the amydgalohippocampal area (C). The midbrain is free of PH3 signal (C and D). AHi: amydgalohippocampal area; Aq: aqueduct; BLA: basolateral amygdala; CA1: field CA1 of the hippocampus; CA2: field CA2 of the hippocampus; CA3: field CA3 of the hippocampus; Mam: mammillary body; MG: medial geniculate nucleus; SC: superior colliculus; SNR: substantia nigra, reticular part. Scale bar = 300 μm for A and D, 600 μm for B and C. (TIF) [file pone.0077710.s004.tif]

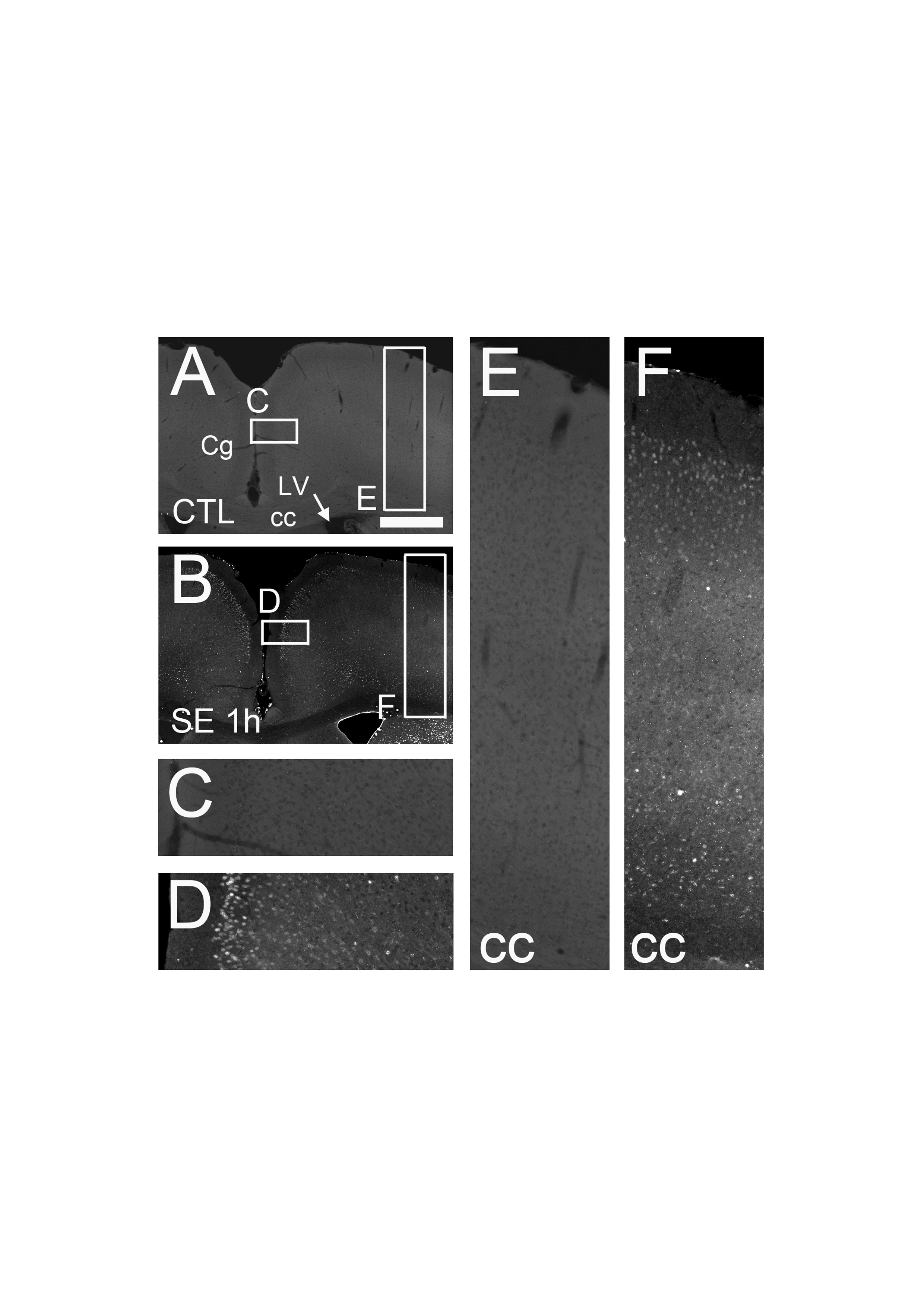

Supplement: Figure S5 — PH3+ cells in the cortex of the SE brain at 1 h. In the cortex of the control brain, there are virtually no PH3+ cells (A, C and E). In contrast, in the upper and lower layers of the neocortex and Cg, there are many PH3+ cells in the SE brain at 1 h (B, D and F). Stacked epifluorescence microscopy images showing the Cg (C and D) and primary motor cortex E and F). cc: corpus callosum. Scale bar = 600 μm for A and B, and 150 μm for C-F. (TIF) [file pone.0077710.s005.tif]

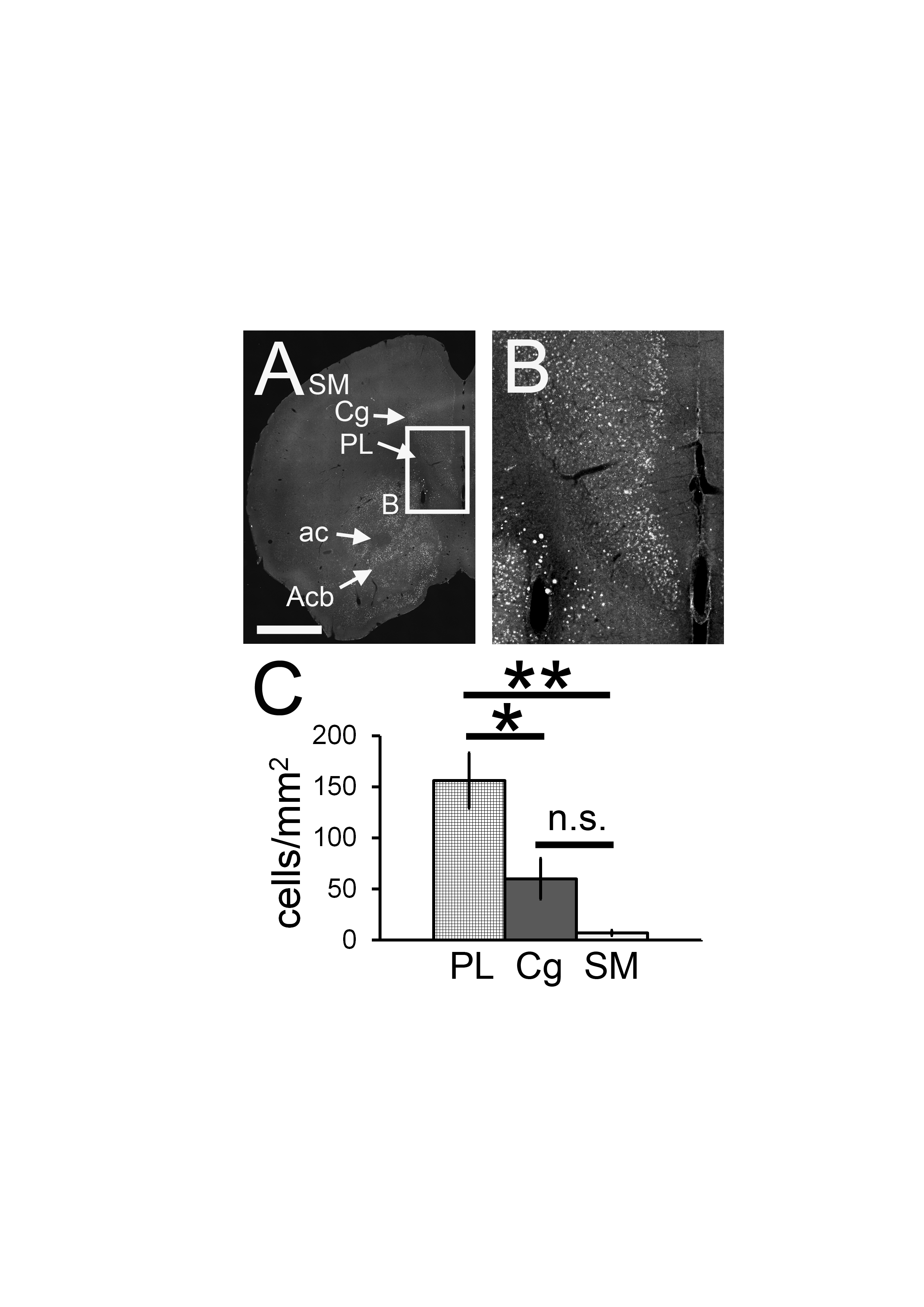

Supplement: Figure S6 — The density of PH3+ cells is different between cortical areas. Stacked epifluorescence microscopy images showing PH3+ cells in the prelimbic cortex 1h after SE induction (A and B). A higher magnification image of the boxed area in A is shown (B). (C) The density of PH3+ cells in the PL is significantly higher than the Cg and sensorimotor cortex. Three animals in each group were analyzed (n = 3). One-way ANOVA followed by Tukey’s post hoc test, * p < 0.05, ** p < 0.01. n.s., not significant. PL: prelimbic cortex; SM: sensorimotor cortex. Scale bar = 1200 μm for A and 300 μm for B. (TIF) [file pone.0077710.s006.tif]

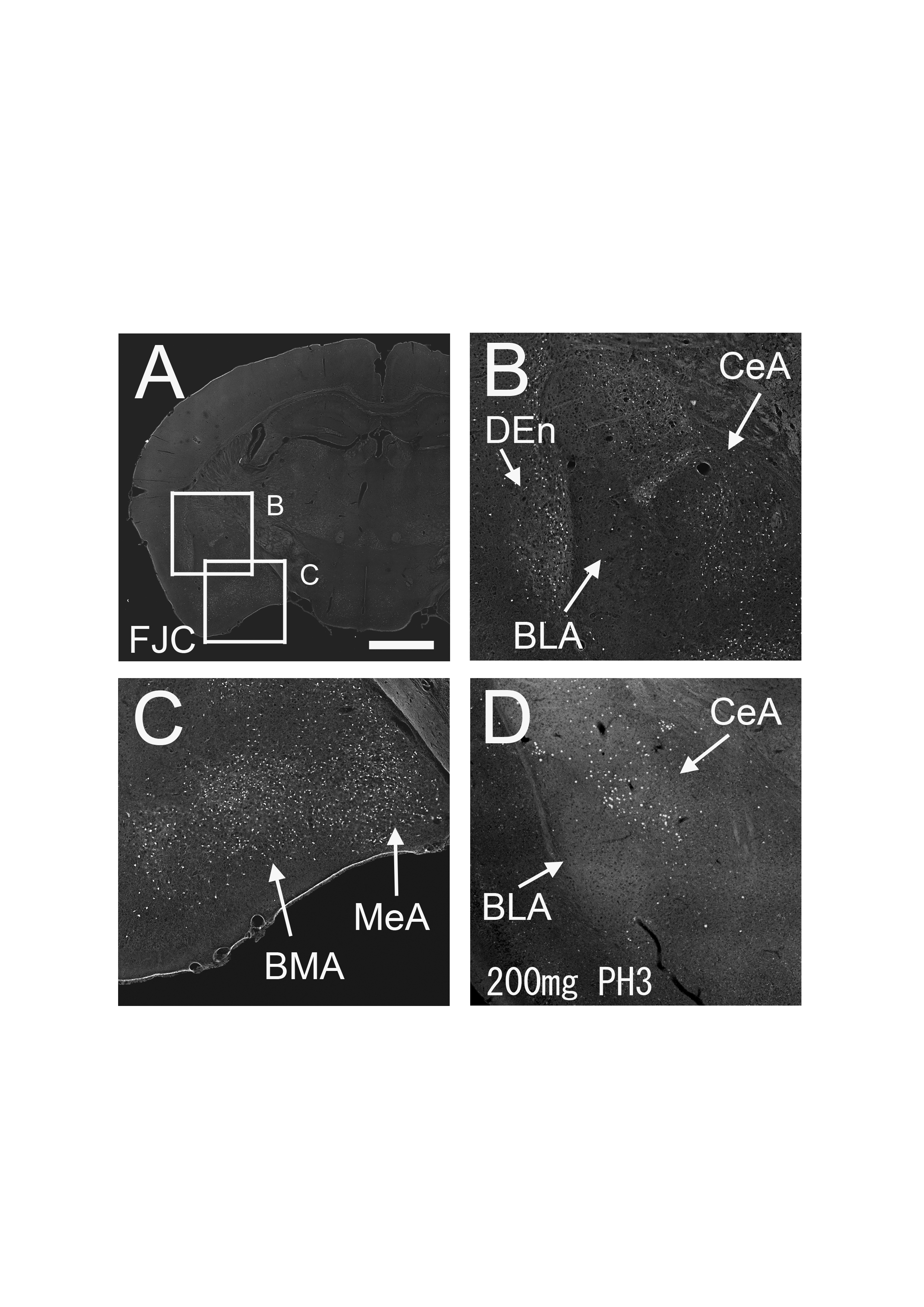

Supplement: Figure S7 — Damaged neurons are detected in the SE brain. Stacked epifluorescence microscopy images showing Fluoro-Jade C (FJC)+ neurons 8 h after SE induction (A-C) and PH3+ cells 1 h after administration of a subconvulsive dose of pilocarpine (200 mg/kg) (D). (B and C) In the amygdaloid complex, many FJC+ neurons are detected, but there are relatively less FJC+ neurons in the BLA. Higher magnification images of the boxed areas in A are shown. (D) Substantial number of PH3+ cells was detected in the CeA at a subconvulsive dose of pilocarpine. BMA: basomedial amygdala; MeA: medial amygdala. Scale bar = 1200 μm for A, 300 μm for B, C and D. (TIF) [file pone.0077710.s007.tif]

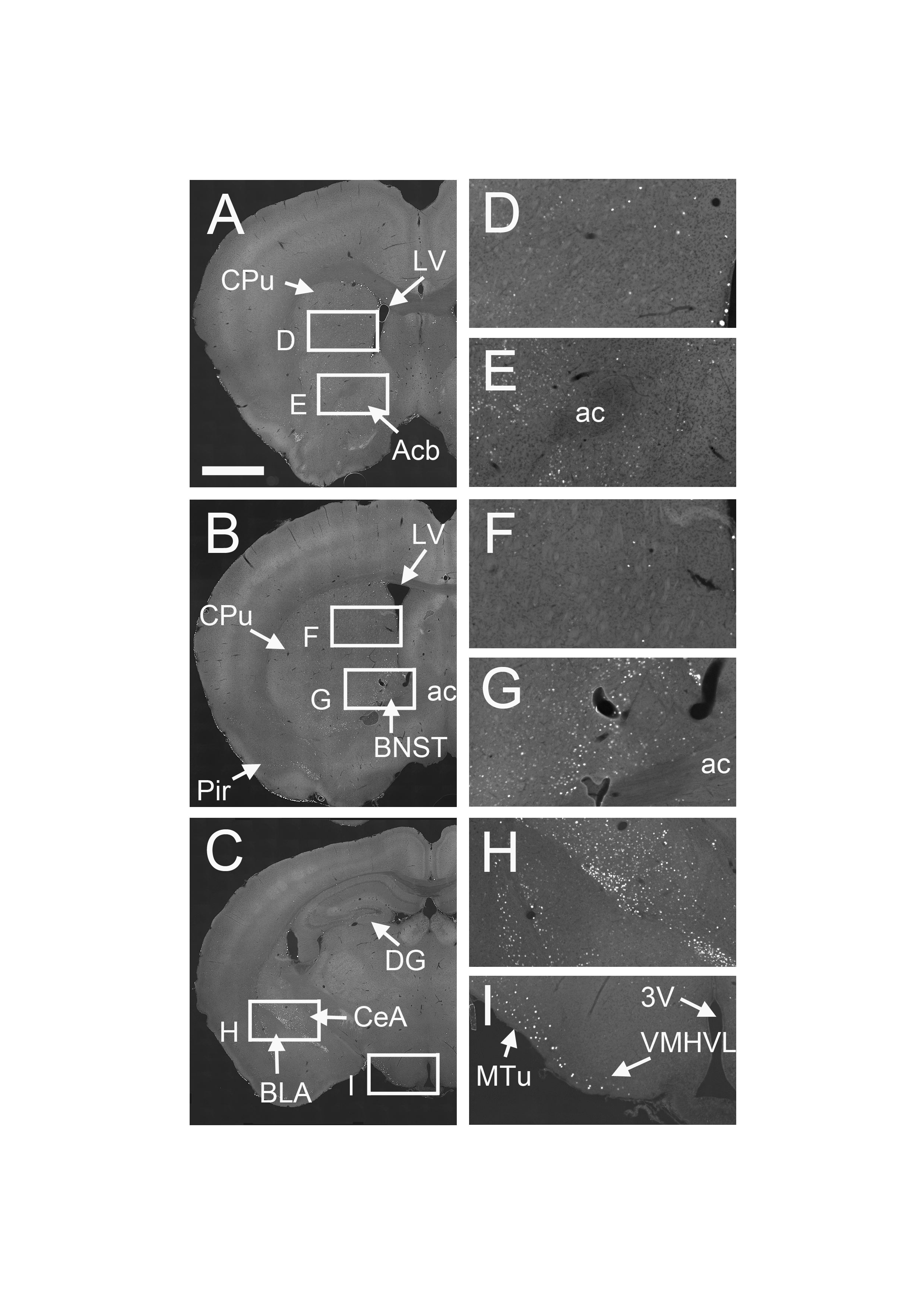

Supplement: Figure S8 — Effect of the NMDA receptor antagonist, MK-801. MK-801 was administered immediately after the onset of generalized clonic seizures and analysis was performed 1h after MK-801. Stacked epifluorescence microscopy images showing PH3+ cells. High magnification images of the boxed areas are shown in each image. In the Acb and CPu, the number of PH3+ cells decreases dramatically (A, B, D, E and F). But there are still non-negligible number of PH3+ cells in the specific structures including the BNST, CeA, MTu and VMHVL (C and G-I). Scale bar = 1200 μm for A-C, 300 μm for D-I. . (TIF) [file pone.0077710.s008.tif]

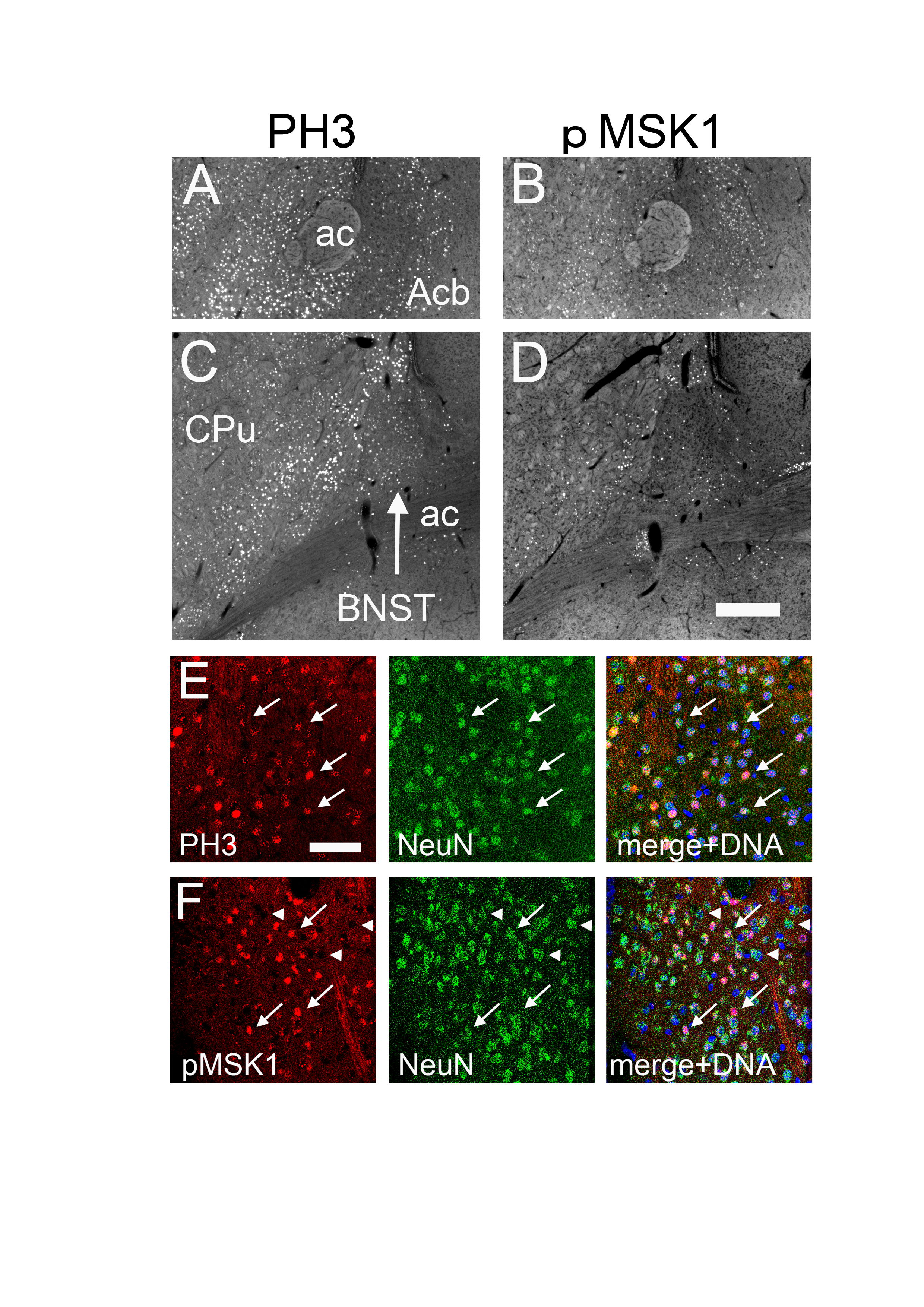

Supplement: Figure S9 — Distribution of pMSK1+ cells is similar to that of PH3+ cells. (A-D) The distributions of PH3+ cells and pMSK1+ cells were compared by using serial sections. Stacked epifluorescence microscopy images are shown. The distribution of pMSK1+ cells is similar to that of PH3+ cells in the Acb, CPu and BNST, but the number of pMSK1+ cells is fewer than that of pH3+ cells (see also Figures 3, 5 and S5). (E and F) Single optical confocal microscopy images of the BNST are shown. Virtually all the NeuN+ mature neurons are PH3+ (arrows, E), but pMSK1+ neurons are a subpopulation (arrows, F). Arrows and arrwoheads indicate NeuN+/pMSK1+ (double labeled) cells and NeuN+/pMSK1- (single labeled) cells respectively. Nuclei were stained in blue with Hoechst33258. Scale bar in D = 300 μm for A-D, scale bar in E = 50 μm for E and F. (TIF) [file pone.0077710.s009.tif]
